# New Mechanical Fat Separation Technique: Adjustable Regenerative Adipose-tissue Transfer (ARAT) and Mechanical Stromal Cell Transfer (MEST)

**DOI:** 10.1093/asjof/ojaa035

**Published:** 2020-07-22

**Authors:** H Eray Copcu, Sule Oztan

**Affiliations:** Department of Plastic and Reconstructive Surgery, MEST Medical Services, Izmir, Turkey

## Abstract

**Background:**

Adipose tissue is not only a very important source of filler but also the body’s greatest source of regenerative cells.

**Objectives:**

In this study, adipose tissue was cut to the desired dimensions using ultra-sharp blade systems to avoid excessive blunt pressure and applied to various anatomical areas—a procedure known as adjustable regenerative adipose-tissue transfer (ARAT). Mechanical stromal cell transfer (MEST) of regenerative cells from fat tissue was also examined.

**Methods:**

ARAT, MEST, or a combination of these was applied in the facial area of a total of 24 patients who were followed for at least 24 months. The integrity of the fat tissue cut with different diameter blades is shown histopathologically. The number and viability of the stromal cells obtained were evaluated and secretome analyses were performed. Patient and surgeon satisfaction were assessed with a visual analog scale.

**Results:**

With the ARAT technique, the desired size fat grafts were obtained between 4000- and 200-micron diameters and applied at varying depths to different aesthetic units of the face, and a guide was developed. In MEST, stromal cells were obtained from 100 mL of condensed fat using different indication-based protocols with 93% mean viability and cell counts of 28.66 to 88.88 × 10^6^.

**Conclusions:**

There are 2 main complications in fat grafting: visibility in thin skin and a low retention rate. The ARAT technique can be used to prevent these 2 complications. MEST, on the other hand, obtains a high rate of fat and viable stromal cells without applying excessive blunt pressure.

**Level of Evidence: 4:**

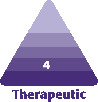

Fat grafting operations are perhaps among the oldest applications of plastic surgery; the first publication was made in the late 19th century.^1^ Similarly, the regenerative effect of adipose tissue was presented by Morestine^2^ in 1915 as the promoting effect of adipose tissue in the healing of the wounded soldiers in World War I. Many studies are available in the existing literature. Studies related with fat grafting deal basically with 3 main topics—fat harvesting, processing techniques, and application techniques, but it must be recognized that the vast majority of this research relates to the pathophysiology, retention, clinical success, and complications of grafting. One of the most important elements affecting all these issues is the size of the fat grafts.^3^ These studies deal basically with 3 main topics—fat harvesting, processing techniques, and application techniques, but it must be recognized that the vast majority of this research relates to the pathophysiology, retention, clinical success, and complications of grafting. One of the most important elements affecting all these issues is the size of the fat grafts.^3^ Recently, Tonnard et al,^4^ who changed our perspective on fat grafts, offered a definition of nanofat in their 2013 study: To work even more superficially with still finer sharp needles (27 gauge), the harvested fat was mechanically emulsified and filtered until a liquid suspension was obtained. They called this “nanofat.” ^4^

Although the term nanofat has become popular since 2013, this new concept has been criticized in 2 aspects: First, in terms of the concept of “nano,” a nanometer is 1-billionth of a meter, and dimensions between approximately 1 and 100 nanometers are known as the nanoscale, but in general,^5^ nanofat is thought of as fat parcel sizes of 600 microns.

Second, in terms of “fat,” Stuzin,^6^ who discussed Tonnard et al’s paper, stated that the most intriguing point in the article is that the substance the authors term nanofat is not fat at all. After histologic examination of their suspension, the authors realized that in processing the nanofat, the normal fat structure was destroyed.^6^ Interestingly, Gustav Neuber^1^ was first to highlight the size of the fat graft in applications with a very long history and also published the first research. He described the fat particles he used as “bean or almond size.” ^1^ Similarly, Peer^7^ used the term “walnut-sized grafts” for adipocyte size in his work published in 1956. To date, more than 50 publications have dealt with the size of the fat graft, and none of them have a clear, mathematical, objective description.^3^ The size of the applied fat tissue is very important from 2 aspects. First, it will determine success, that is, retention of the fat graft.^3^ As the diameter of adipose lobules increases, the central zone of necrosis will theoretically expand according to diffusional limitations. Therefore, one can see that the size of fat particles may ultimately influence how much of the grafted material survives.^8^ Second, the adipocyte diameter is very important for the aesthetic result. Skin thickness and subcutaneous fat tissue density and thickness are not the same in every part of the body, and fat grafts should be applied according to the original structure of the anatomical area. Fat grafting should be applied in different aesthetic units and different diameters and depths, especially in the face area, just like hyaluronic acid (HA) applications. The search for a decisive tool for the particle diameter of adipocytes is ongoing. Tonnard et al’s emphasis on “nanofat” is its regenerative effect.^4^ In fact, one of the most important features of fat tissue, in addition to its thermoregulation, shock absorption, and being the body’s energy storage, is that it is the largest and most important regenerative cell source in the body.^9^ There are 2 types of cells in all organs that make up our body—parenchymal cells responsible for the function of the organ and stromal cells that support them. Stromal cells provide tissue repair and renewal following stress, injury, illness, or aging, that is, from both intrinsic and extrinsic causes.^10^

Until recently, the gold standard was the use of enzymes in the extraction of stromal cells from adipose tissue. The enzyme destroyed the dense bonds in the adipose tissue, and stromal cells were obtained by centrifugation.^11^ This stromal cell cocktail, including fat stem cells, was called stromal vascular fraction (SVF). Regulatory authorities evaluated SVF production as biological drug production and required that current good manufacturing practice (cGMP) and current good laboratory practices (cGLP) standards be met.^12^ More importantly, in the production of SVF by enzyme, the enzyme destroys not only bonds but also extracellular matrix (ECM) and regenerative cells.^13^ For these reasons, mechanical stromal cell production has become increasingly popular.^14,15^ Recent study by Sesé et al^16^ has shown that stromal cell extraction by mechanical means yields not only high quality and a diversity of cells but also 10 times greater cell counts. In this study, patented sharp blade systems (Adinizer, BSLrest, Busan, South Korea); with patented protocol (IPs: Indication based protocols, patented by Eray COPCU), fat tissues were cut without killing adipocytes and without creating excessive blunt pressure; thus, varying diameters of adipocytes were obtained for different anatomic areas. In addition, stromal cells separated after these processes were obtained by centrifugation and used for regenerative purposes.

## METHODS

This study was conducted in accordance with the principles of the Declaration of Helsinki. All patients were provided detailed information preoperatively and gave written consent for all surgical procedures, anesthesia, intraoperative video recording, and photography. In addition, a written consent form was obtained from the patients stating that they willingly donated their adipose tissue for laboratory analysis.

In this study, a patented CE marking and ISO 13485-certified blade system was used, and rules of minimal manipulation were followed. No enzymes and similar chemicals were used, and the structure of the fat tissue was not altered. All sterilization protocols were followed, and all procedures were performed in a sterile operating room environment. Thus, IRB approval was not required for this study.

The adjustable regenerative adipose-tissue transfer (ARAT) and the mechanical stromal cell transfer (MEST) techniques we described were applied to 24 consecutive patients who were followed minimum 24 months between August 2017 and March 2020 were included this study. We did not have exclusion criteria for the participation in this study. Adipose tissue was cut with a sharp blade system (Adinizer) between 4000 and 100 microns without blunt pressure. The integrity of the cell at each stage was evaluated histopathologically with hematoxylin-eosin dye. The final products containing stromal cells obtained were evaluated simultaneously with a LunaStem device in the operating room environment for viability and cell number. Final products were transferred to a sterile bag and transported to the cell therapy unit for further analysis. Extensive characterization tests were performed to obtain data from the adinized products of adipose tissue, and the following analyses were performed on different regenerative products.

### Directly on Adipose Tissue

#### Secretome

Non-washed adipose tissue (NWAT) and emulsified fat from Adinizer (500 μL) were placed in a 48-well collagen-coated plate (CellAffix, APSciences, Columbia, MD, USA) and incubated for 30 minutes at 37°C with 5% CO_2_ to allow contact with collagen. After this incubation, 500 μL of non-supplemented Dulbecco’s Modified Eagle Medium (DMEM; Gibco, Thermo Fisher Scientific, Waltham, MA, USA) was added to the mixture and incubated for 24 hours at 37°C with 5% CO_2_. After 24 hours, the samples were centrifuged (Multifuge Heraeus 3 S-R centrifuge, Thermo Scientific, Indianapolis, IN, USA) at 1500 rpm for 5 minutes to remove fat, and liquid samples were stored at −80°C until used for analysis. A combination of 11 cytokines and growth factors (vascular endothelial growth factor, interleukin-1 receptor antagonist, fibroblast growth factor 2, interleukin-1β, transforming growth factor β1, platelet derived growth factor aa-bb or ab-bb, epidermal growth factor, interferon-γ, interleukin-6, interleukin-10, and tumor necrosis factor-α) were measured using a Magpix instrument (Luminex xMAP Technology, Luminex Inc., Austin, TX, USA) allowing simultaneous measurement of the different analytes in a small sample volume.

#### Culture

About 0.5 to 1 mL of NWAT and emulsified fat from Adinizer were placed in a T25 flask with the minimum amount of proliferation culture media to favor mesenchymal stem cells adhesion directly from adipose tissue. Culture was checked every 2 days and trypsinization was performed as soon as mesenchymal stem cells were confluent in one of the 2 flasks. Viability and total of viable nucleated cells obtained were measured using the LunaStem Automated Fluorescence Cell Counter device (Logos Biosystems, South Korea).

### After SVF Extraction

#### Viability and Nucleated Cell Counts

Total viable nucleated cell (NC) recovery and viability percentage were determined using the LunaStem Automated Fluorescence Cell Counter device (Logos Biosystems, South Korea).

#### CFU-F Assay

Clonogenic potential of mesenchymal stem cells from SVF was assessed using colony forming unit-fibroblast (CFU-F) assay as follows: 500 SVF cells were seeded in 6-wells plate in triplicate in proliferation medium (DMEM/Ham’s Nutrient Mixture F12/fetal bovine serum 10%, Glutamax, Gentamicin, Penicilline G, Fungizone). Colonies were grown for 10 to 14 days, depending on the growth rate of the cells. At the end of the assay, the culture dishes were rinsed with phosphate-buffered saline twice and then fixed with 10% neutral-buffered formalin for 20 minutes and then stained with 0.5% crystal violet (Sigma-Aldrich, Saint-Quentin-Fallavier, France). Cell colonies were counted using phase-contrast microscopy. All 3 Petri dishes were counted, and the average and standard deviation were calculated to generate the final frequency percentage value expressed as colony-forming efficiency by dividing the number of cells seeded × 100.

#### Flow Cytometry Analysis of SVF Cell Subsets

Characterization of the SVF cell subpopulations was performed according to the recommendations of the International Federation of Adipose Therapeutics and Sciences (IFATS) and the International Society for Cellular Therapy (ISCT). Briefly, SVF suspension was digested with DNase I 10U/mL (Roche Diagnostics, Indianapolis, Indiana, USA) in Dulbecco’s Phosphate Buffered Serum (DPBS) Ca^++^/Mg^++^ free medium containing 0.1 mM ethylenediaminetetraacetic acid, 25 mM Hepes, 1% fetal calf serum for 15 minutes at 37°C and filtered through a 70-μm nylon cell strainer to eliminate the majority of cell aggregates. Cells were centrifuged, resuspended, and labeled 20 minutes at 4°C with the following fluorochrome-conjugated antibodies: CD14-FITC, CD90-FITC, CD146-PE, CD34-ECD, CD45-PC5 (Beckman Coulter, Miami, FL, USA) or their isotype control to determine nonspecific fluorescence. Red blood cells were lysed in NH_4_Cl for 10 minutes at 4°C before cells were centrifuged and resuspended in DPBS Ca^++^/Mg^++^. NucBlue (Thermo Fisher Scientific, Waltham, MA, USA), allowing discrimination of viable and dead cells, was added for 5 minutes before flow cytometry analysis on a NAVIOS flow cytometer (Beckman Coulter). CD45 negative cells were discriminated in CD34^−^CD146^+^ and CD34^+^CD146^−^CD90^+^ described as regenerative perivascular cells, and CD34^+^CD146^+^ as endothelial cells.

### Viability and NC Count in the Operating Room

Total viable NC recovery and viability percentage were determined using the LunaStem device. It uses adipose-derived stem cells and SVF samples to count live NCs, dead NCs, and non-NCs with precision and consistency. For downstream procedures, 2 μL of cells of acridine orange/propidium iodide stain was added to 18 μL stromal cell composition and mixed by pipetting up and down; 10 μL of the mixed cell sample was placed into the inlet of one chamber of the counting slide and counted. The number of total cells and their concentrations (nucleated or non-nucleated), the number of live and dead cells and their concentrations (nucleated), the viability percentage (nucleated), cell images, and a histogram of cell size distribution and size gating were obtained in the operating room. All data were recorded.

### Preparing of ARAT and MEST

**Figure 1.**
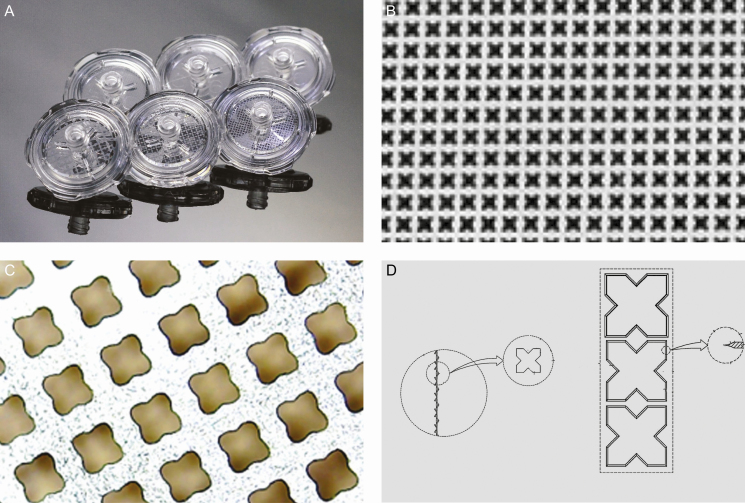
(A) Patented ultra-sharp blade systems (Adinizer) from 4000 microns to 100 microns. (B) Adinizer with 200-micron blades. (C) 500 × magnification of Portion A. (D) Schematic view of cross section of the Adinizer double-sided blades.

Adipose tissue is one of the most fragile and sensitive tissues in the body; obviously, blunt pressure should be avoided, and the tissue should be treated with maximum sensitivity to achieve the best results. Our approach was called the gentle touch system (GTS). We used a patented very sharp blade system in different diameters, while both bringing the adipocytes to the size we wanted and in mechanically obtaining stromal cells from adipose tissue (Figure 1). In the ARAT procedure, there are 4 basic steps; adding MEST with a final centrifugation is the fifth. Each step has its own philosophy.

### ARAT Procedure

#### Tumescent Application

**Figure 2.**
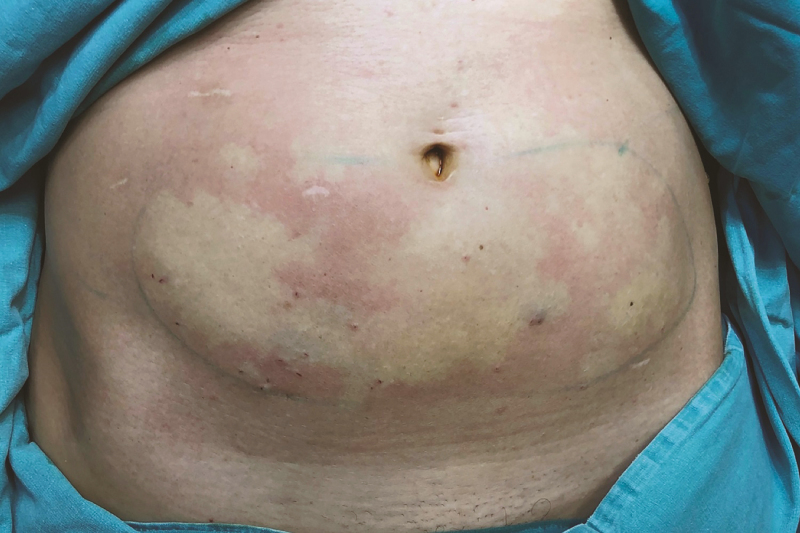
Blanching effect of adrenaline in a 57-year-old female patient. This effect is the indicator for the effective vasoconstriction.

An important step in obtaining effective stromal cells is the use of high quality, least traumatized fatty tissue containing the fewest blood elements. This is crucial for correct and effective tumescent applications. If the procedure is performed under general anesthesia, no local anesthetic is added to the tumescent fluid; 2 mg of adrenaline in 1000 mL of saline was applied to the donor area. The 2 most important elements here are first, to use wet technique instead of super wet; in other words, tumescence should be given up to the lipoaspirate to be taken, and second, the effect of adrenaline must be awaited patiently for at least 15 minutes. Adrenaline must be stored without light, and to avoid any loss of effect, the ampoule of adrenaline should be placed in the tumescent fluid immediately after breaking and applied to the tissue without delay. An indicator of the adequacy of the process is seeing a blanching effect of the adrenaline on the tissue surface after applying the tumescent fluid (Figure 2).

#### Lipoharvesting

Adipose tissue responds to pressure by cell death; therefore, excess pressure should be avoided in both lipoharvesting and lipofilling processes. In the lipoharvesting step, a modification of the cannula developed by Dr Delay (Delay cannula, PLA 87, Pouret Medical, Paris, France) was used for minimum pressure and maximum attention to tissue. We used a 2.8-mm diameter cannula with a blunt tip and 4 eccentric holes. The fat tissue was manually harvested using 10/20/60 mL BD injector luer-lock.

#### Lipo-Condensation

Condensation procedures aim to increase the relative number of stromal cells per tissue volume simply by eliminating some components, such as adipocytes, red blood cells, oil, and aqueous fractions, which are present in the lipoaspirate. The principal methods for adipose tissue condensation are gravity-based decantation, filtration, and centrifugation.^17,18^ For our purposes, the centrifuge method was preferred. A different piston and plunger system was used to easily transfer the products obtained after the procedure. For condensation, lipoaspirate was centrifuged at 500*g* for 2 minutes, and the tumescent and blood elements remaining in the lowest layer were discarded.

#### Adinizing

**Figure 3.**
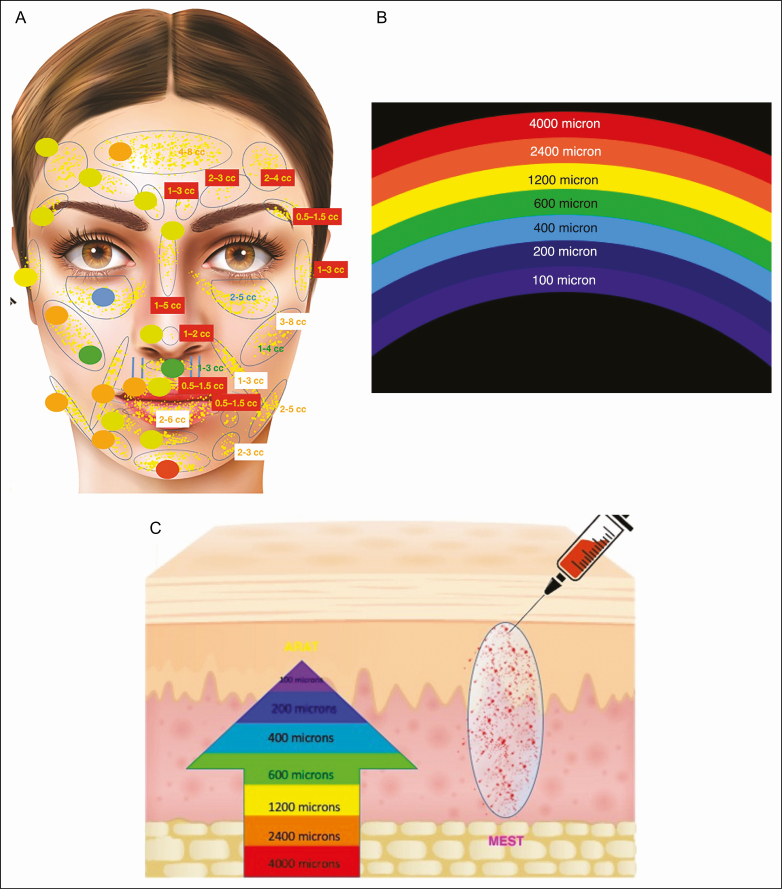
(A) Guide for adjustable regenerative adipose transfer (ARAT) on the face. Each color represents the thickness for each. Average amounts were shown in milliliters. In the model we developed and presented in this figure, the image on the right face shows the application to different areas of the face in accordance with the concept of compartments defined by Rohrich and Pessa, and the image on the right face shows the application of different size fat grafts to different depths defined by Cohen. (B) Rainbow approach: different colored (diameter) Adinizers for different depths of the face. Each color represents a different diameter. (C) Application of adinized adipose tissue.

There is no term in the literature that describes the process of cutting fat tissue with blades. “Adinizing” is a new term and means cutting the fats with ultra-sharp blades to bring the fat tissue to the desired size and to separate the stromal cells from the parenchymal cells in adipose tissue. Patented Adinizer blade wheels are used for this process. These start from 4000 microns and alternate wheels include blades of 2400, 1200, 600, 400, 200, and 100 micron sizes. The use of these wheels is indicated on a rainbow scale. The most important precaution in GTS is to treat the adipose tissue as gently as possible. Starting the adinizing process with a small diameter knife will increase the amount of blunt trauma or positive pressure per unit of adipose tissue. Therefore, the adinizing process starts with 4000 microns—the red smart wheel—and decreasing diameters are used in descending order through the blades with 2400, 1200, 600, 400, 200, and 100 micron diameters, to ensure that the minimum pressure is applied to the adipose tissue. If the adinizing is to be done for the application of ARAT, that is, for fat grafting, adipocytes of the desired diameter are obtained by using condensed fat and 10 or 20 mL luer-lock injectors. The luer-lock inlets on both ends of the smart wheel are fitted with an injector with condensed fat on one end and an empty luer-lock injector on the other end. The pistons of the 2 injectors are moved back and forth at a speed of 10 mL/sec to cut the adipose tissue with the blades in the smart wheel and reach the desired diameter. In order not to apply blunt pressure to the adipose tissue, the adinizing process must be done in order, from large to small diameter. Adipose tissues obtained in different sizes can be applied to different anatomical areas and depths. A guide has been created for face applications (Figure 3).

### MEST Procedure

For MEST, after the above steps, centrifugation is done to obtain stromal cells. In mechanically obtaining stromal cells from adipose tissue, in all techniques described to date, the stromal cells are in a dense layer as solid clusters, usually gel or stromal aggregates.^19-21^ However, the final product in SVF production with enzyme is a solution-form cocktail, and it is easy to apply. A new protocol has been defined and patented to obtain stromal cells with different compositions and physical structures for various indications in mechanical techniques—indication-based protocols (IPs) (Table 1).

**Table 1.** Summary of Indication-Based Protocols

IPs no.	Total volume (mL)	Suggested fat volume (mL)	Suggested saline volume (mL)	Desired product	Desired volume (mL)
1	10	5	5	Solution	5-6
2	20	10	10	Solution	8-12
3	10	10	0	Gel/dense solution	1-2
4	20	20	0	Gel/dense solution	3-4

In this protocol, we developed varying amounts of stromal cells for different indications, especially in the solution form. Four separate IPs were defined. The aim is the isolation of the bottom stromal cell solution (SCS) and the cell aggregate, and the final product, obtained by mixing these 2 layers together, is called the total stromal (TOST) fraction or cells.

Adipose tissue was prediluted with saline using 10 or 20 mL injectors in IPs 1 and 2, while condensed adipose tissue was used directly in IPs 3 and 4. In accordance with the selected IPs, 10- or 20-mL luer-lock injectors were used. An injector with condensed fat was placed on one end of the Adinizer disk, an empty injector was placed on the other end, and the fat tissues were cut with sharp blades in the Adinizer under minimal pressure with back and forth movements. The back and forth movement was made 25 times on average (range, at least 20 and up to 30 times). While starting the process of mechanically cutting the fat, pressure can be felt in the injectors between the researcher’s fingers, and this pressure subsides after approximately 20 passes. The relief of the pressure indicates that sufficient cuts have been made, and the process is terminated with a small cut having been made. Adinizing was first performed with a 4000-micron Adinizer; after approximately 25 passes, the cutting process was continued with the next-smaller diameter disk. After each adinizing, a sample was taken and histopathological examination was performed to examine the fat tissue.

#### Stromal Cell Isolation

**Figure 4.**
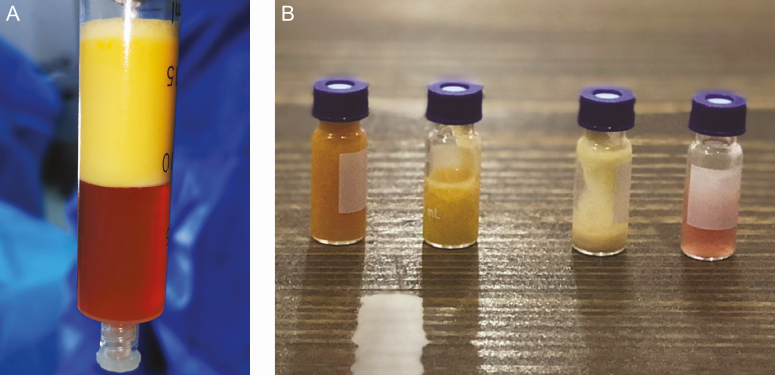
(A) Final product after mechanical stromal cell isolation. (B) There are 4 layers after the mechanical stromal cell process. From left to right: triglycerides (TGs), adipocytes, stromal cell aggregates (SCA), and the stromal cell solution (SCS).

After the adinizing process, the product is centrifuged for the last time at 1200*g* for 6 minutes, and as a result of this process, 4 layers are obtained depending on the density. These layers are SCS at the bottom, stromal-cell aggregate (SCA) above it, adinized fat tissue layer above that, and triglycerides at the top. The bottom layer and just above SCS and SCA were taken for stromal cell transfer and used for regenerative purposes. The determination of TOST (total stromal) cell was used for these 2 plates (Figure 4). The above steps and ARAT/MEST procedures are presented in the videos (Videos 1-3).

#### Data Acquisition and Processing

The patients were verbally informed about the sample to be taken. All patients took standard photography preoperatively and postoperatively at a minimum of 3 time-points (6 months, 1 year, and 2 years postoperative, respectively) (Supplemental Figures 1-3, available online at www.asjopenforum.com).

## RESULTS

The mean time of follow-up was 26 months (range, 24-30 months). The average age of the patients was 47 years (range, 14-82 years). Nineteen (79.16%) of the cases were female and 4 (20.84%) were male. The features and visual analog scale (VAS) results of the cases are presented in Table 2.

**Table 2.** Features and VAS Results of Patients

			Patient	VAS scores		Surgeon	VAS scores	
Sex	Age	Operation	6th month	1 year	2 years	6th month	1 year	2 years
M	24	MEST	8	7	7	7	8	8
F	36	MEST	7	7	8	7	7	7
F	38	Combined	8	8	7	9	8	8
F	82	Combined	9	9	8	8	8	8
F	67	Combined	8	9	7	8	8	7
F	56	Combined	7	8	8	8	8	8
M	49	Combined	7	7	7	8	8	8
M	24	MEST	8	9	9	8	9	9
F	55	MEST	9	8	8	8	8	8
F	28	Combined	9	8	8	9	8	8
F	42	MEST	8	7	8	7	7	7
F	41	Combined	8	7	6	7	7	7
F	57	Combined	7	8	8	8	8	8
F	65	Combined	7	7	9	8	7	8
F	63	ARAT	6	7	7	6	6	6
F	21	MEST	8	9	9	8	8	8
M	69	Combined	8	7	6	6	6	7
F	14	Combined	9	8	8	8	7	6
F	56	Combined	8	8	7	7	7	8
F	43	ARAT	7	6	6	6	6	6
F	49	MEST	9	8	8	8	7	7
F	58	MEST	9	8	8	8	8	8
M	58	ARAT	8	7	7	7	7	7
F	32	MEST	8	8	8	8	8	8

ARAT, adjustable regenerative adipose-tissue transfer; MEST, mechanical stromal cell transfer; VAS, visual analog scale

**Figure 5.**
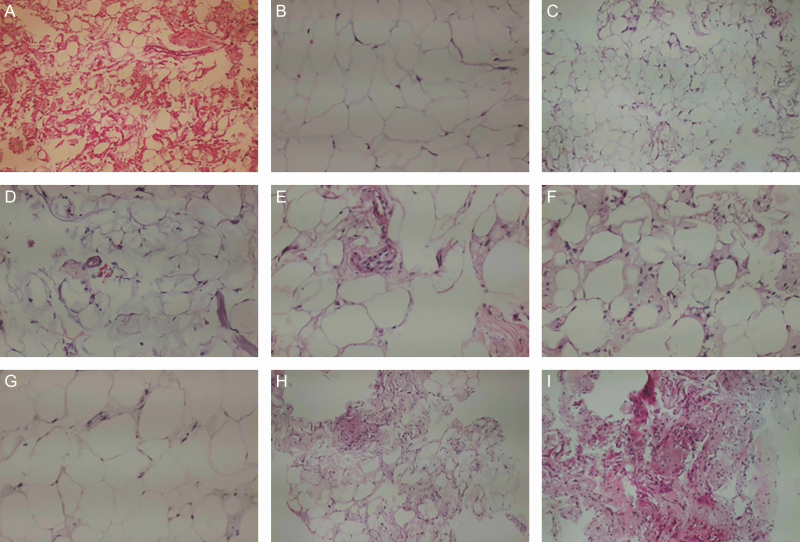
Histopathological assessment of lipoaspirate, adinized adipose tissue, and stromal vascular fraction (SVF) with hematoxylin-eosin staining. (A) Lipoaspirate: there are many different types of cells. (B) Condensed fat after the first centrifuge. There are pure adipocytes. Adipose tissue after (C) 4000-micron adinizing, (D) 2400-micron adinizing, (E) 1200-micron adinizing, (F) 600-micron adinizing, (G) 400-micron adinizing, and (H) 200-micron adinizing. Intact adipocytes could be seen after all adinizing sessions. (I) Mechanical SVF. All kinds of cells can be seen in total stromal (TOST)-cells except only adipocyte.

In the ARAT procedure, in accordance with the guide in Figure 3, a minimum of 22 mL to a maximum of 68 mL adipose tissue was applied to the aesthetic units of the face. None of the cases had complications during the preoperative stage or postoperatively. Especially in applications in areas with the thinnest skin of the body, such as the periorbital area, no complications, such as the visibility of the fat tissue, were observed early or late in the postoperative period. While preparing the ARAT/MEST technique, the samples taken after adinizing were evaluated histopathologically by hematoxylin-eosin staining; the results are presented in Figure 5. In the ARAT procedure, different diameter fat grafts were chosen in accordance with the application depth. While the thickest grafts are applied directly on the periosteum, smaller-diameter grafts are applied more superficially. In the periorbital area, 200/400 micron size adiposities can be safely applied. An IP is used in MEST application with different IPs preferred for each indication.

The volumes of end products containing TOST cells obtained after each IP are variable; these volumes, cell numbers, and viability rates are presented in Table 3. All cases were evaluated simultaneously in the operating room environment using a LunaStem dual fluoroscopy device for acridine orange/propidium iodide dyes. In addition, total flow cytometric cell numbers and viability were measured cytometrically; values for each IP are presented in Tables 4 and 5. When performing secretome tests, VEGF-A, EGF, FGF-2 PDGF, nerve growth factor [NGF], TGFB1 were assessed in the regenerative group; interferon gamma (IFNg), IL-1b, IL, and TNFa were evaluated in the anti-inflammatory group; and IL-10 and IL-1ra in the inflammatory group; the results are presented in Table 6.

**Table 3.** Comparison of IPs for Total Volume and End-Product

IPs	Condensed fat volume (mL)	Pre-adinizing total volume (mL)	Post-adinizing total volume (mL)	TOST-cell volume
IPs1	5	10	9	4.8 ± 1.2
IPs 2	10	20	19	10.1 ± 1.8
IPs 3	10	10	8	1.8 ± 0.6
IPs 4	20	20	18	3.9 ± 1.7

IPs, indication-based protocols; TOST, total stromal.

**Table 4.** Viability and Total Number of Nuclear Cells Per Milliliter, Total NCs, and 100 mL After Adinizing for Each IPs by Dual Fluoroscopy Device

IPs	Viability %	NCs (average) 10^6^ per mL	Total NCs (average) 10^6^	100 mL condensed fat Total NCs 10^6^
IPs1	94 ± 2	0.81	3.88	77.76
IPs 2	93 ± 2	0.74	7.74	74.74
IPs 3	93 ± 2	1.41	2.54	25.4
IPs 4	91 ± 4	1.26	4.91	24.57

IPs, indication-based protocols; NCs, nuclear cells.

**Table 5.** Viability and Total Number of Nuclear Cells Per Milliliter, Total NCs, and 100 mL After Adinizing for Each IPs by Flow Cytometry

IPs	Viability %	NCs (Average) 10^6^ per mL	Total NCs (average) 10^6^	100 mL Condensed Fat Total NCs 10^6^
IP's1	94 ± 2	0.91	4.37	87.36
IP's 2	93 ± 2	0.88	8.89	88.88
IP's 3	93 ± 2	1.61	2.9	28.98
IP's 4	91 ± 4	1.47	5.73	28.66

IPs, indication-based protocols; NCs, nuclear cells.

**Table 6.** Analysis of Secretomes in pg/mL

Regenerative	[VEGF-A]	43.52± 12.21
	[EGF-A]	16.44 ± 2.67
	[FGF-2]	8519.31 ± 3122.42
	[PDGF]	64.60 ± 21.43
	[NGF]	26.12 ± 14.78
	[TGFB1]	840.94 ± 115.77
Anti-inflammatory	[IL-10]	246.77 ± 116.86
	[IL-1ra]	417.21 ± 211.37
Inflammatory	[IFNg]	2.20 ± 1.85
	[IL-1b]	1221.44 ± 664.37
	[IL-6]	17338.21 ± 3224.60
	[TNFa]	68.12 ± 21.44

EGF, epidermal growth factor; FGF, fibroblast growth factor 2; IFNg, interferon gamma; IL-10, interleukin 10; IL-1b, interleukin 1 beta; IL-1ra, interleukin-1 receptor antagonist; IL-6, interleukin 6; NGF, nerve growth factor; PDFG, platelet-derived growth factor; TGFB1, transforming growth factor-β1; TNFa, tumor necrosis factor-alpha; VEGF, vascular endothelial growth factor.

All cases were followed-up for at least 24 months. Patient satisfaction of the surgery was evaluated using a VAS ranging from 1 (least satisfied) to 10 (most satisfied) 3 months after surgery by patients and surgeons. Evaluation, in the ARAT procedure, was made in terms of retention of fat tissue, change in skin quality, and correction of wrinkles; evaluation in the MEST procedure was made in terms of skin quality and texture, tone, luster, and looking younger and fresh.

**Figure 6.**
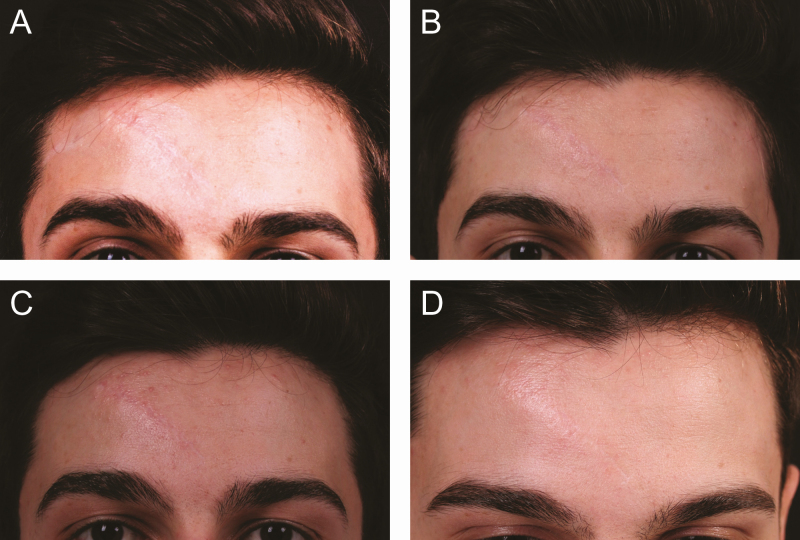
Scar revision in the frontal area with only mechanical stromal cell transfer (MEST) indication-based protocols 1 (IPs1) in a 24-year-old male patient. He had a traffic accident history 1 year before the surgery. (A) Preoperative view and postoperative view at (B) 6 months, (C) 1 year, and (D) 2 years.

**Figure 7.**
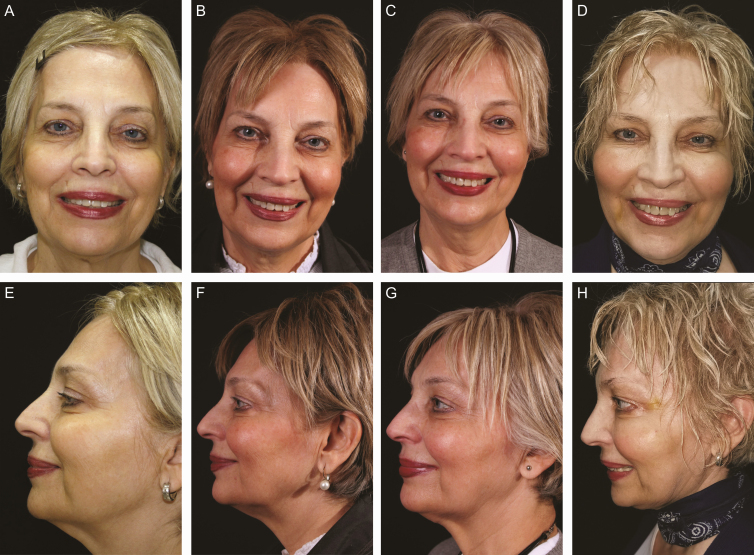
A 65-year-old female patient operated on with the adjustable regenerative adipose-tissue transfer (ARAT) and mechanical stromal cell transfer (MEST) technique indication-based protocols 4 (IPs4). Total of 42 mL fat was used. (A) Preoperative and postoperative frontal view at (B) 6 months, (C) 1 year, and (D) 2 years. (E) Preoperative and postoperative lateral view at (F) 6 months, (G) 1 year, and (H) 2 years.

The results were scored by both patients and surgeons using VAS, and the results are presented in Table 2. In evaluations at the end of 6 months, the patients rated results between 6 and 9 (average, 7.91), and the surgeons gave scores between 6 and 8 (average, 7.58). Similarly, at the end of the first year, the average patient assessment was 7.71 and the surgeons’ was 7.46. Finally, at the end of 2 years, the patients’ average assessment was 7.54 and the surgeons’ was 7.5. The VAS results of the combined applications are higher than those of MEST or ARAT applications alone. Since they were asked to interpret the results, the most common comment was the change in the quality of the skin and improvement in color (Figures 6 and 7).

## DISCUSSION

Adipose tissue is an excellent biological filler because it is readily available, easily obtainable, repeatable, inexpensive, versatile, biocompatible, and has low donor-site morbidity.^8^ However, despite these positive aspects, many problems may be encountered in fat graft applications, such as insufficient retention, visibility, cyst formation, and calcifications.^8^

The concepts of redefining fat graft size and proper anatomical placement were first described by Cohen et al.^22^ Four types of fat grafts were defined according to parcel size, harvesting cannulae, and process. (1) Macrofat: Fat grafts harvested with cannula larger than 2.4 mm, fat grafts larger than 2.4 mm, which can be given without any operation. It is recommended to apply them to the breast, buttock, and trunk. (2) Millifat: Fat grafts harvested cannula with 2.4-mm hole diameter, washing and 2.4 mm emulsifier, and parcel diameter less than 2.4 mm. It is recommended to apply to deep tissue, lips, temporal space, and chin. (3) Microfat: Fat grafts harvested cannula with 1.2- to 2.4-mm hole diameter, 1.2 mm emulsifier, and parcel diameter less than 1.2 mm. It is recommended to apply them on the forehead, eyelid, brow, perioral, periorbital, nose, and hand; and finally (4) Nanofat: Fat grafts harvested with cannula with a diameter of 1.2 to 2.4 mm, 400 to 600 micron emulsifier, and 400 to 600 micron parcel diameter. They recommended the use of nanofat for superficial rhytids, intradermally.^22^

The ARAT technique is different from these 4 fat graft definitions. We use only one type of cannula, and we do not emulsify adipose tissue by applying excessive blunt pressure with a connector or filter. We can obtain the desired net diameter of adipose tissue for the anatomical area we want to apply, using only sharp blades (Figure 1).

Loss or deposition of fat creates a change in the surface topography of the face associated with the perception of facial aging, and the concept of fat compartmentalization was described by Rohrich and Pessa.^23^ Defined anatomical boundaries of the nasolabial, medial, middle, lateral superficial cheek, deep medial cheek, suborbicularis, buccal, and periorbital fat compartments provide evidence of the compartmentalization of the facial soft tissues.^19^ The ARAT technique was performed in the face, in accordance with these compartments and Cohen’s principles: thicker fat graft for deeper and smaller diameters for more superficial depth. In order to make this application better, the rainbow approach, seen in Figure 3, was developed. We use different blades with different colors and diameters.

Although different approaches have been tried in each step of fat grafting, the search for an ideal approach continues. We believe that the use of fat graft applications requires the application of specific thicknesses of grafts for different anatomic areas, as with HA applications. Just as HA fillers have been characterized by their cohesivity, viscosity, and elasticity into different products for different uses, fat can be modified by cleaning, compounding, emulsification, and filtering into specific shapes of the same tissue.^24^

To date, many definitions have been suggested for the size of the fat graft, but none is a mathematical, objective definition.^3^ For the first time, the adipocyte diameter applied in previous studies was objectively determined in this study. Tonnard et al^4^ used the terms macro- and micro-fat to distinguish the fat tissue sizes in his study, but these are related to harvesting, that is, they refer to the harvested fat tissue not the size of the applied fat.

The term adinizing, which we defined above, is used for the first time in the literature; it defines the process of cutting fat tissue without blunt pressure, with sharp blades for reducing grafts to the desired diameter and separating the parenchymal and stromal cells in adipose tissue without using enzymes.

Adipose tissue is a type of loose connective tissue that contains an eclectic reservoir of cells, including immune cells, erythrocytes, progenitors, and stromal components.^25^ The stroma is the area with support cells; there are also adipose-derived stem cells (ASCs) in the perivascular area of the stroma.^4^ The separation process with sharp blades will not only ensure the separation of stromal cells but also ASCs.^13^ When this work is done with an enzyme, such as collagenase, destruction in both ECM and stromal regenerative cells occurs.^13^ Obtaining stromal cells from adipose tissue mechanically has been very popular recently; there are studies in the literature related to many devices.^13,16,18^ Unfortunately, unlike the enzyme, there are no established standards for the mechanical methods. This concerns both the specifications used and the presentation of the results. First, when the enzyme is used, the final product is SVF. The end product obtained mechanically has a different composition than SVF obtained by enzyme; ^16^ therefore, a different definition is needed. We recommend using the term TOST cells for mechanical stromal cells, instead of SVF. Unlike the decomposing enzyme, stromal cells and ECM in mechanical methods are not destroyed and are protected to the maximum. Very different results have been reported regarding the viability and number of cells in mechanical stromal cell production.^4,13,16,26^ In Tonnard et al’s^4^ study, the first research published on this subject, in measurements using flow cytometry, 1,975,000 cells were reported in 100 mL of oil, while the lowest rate was found to be 28,660,000 and the highest 88,880,000 in our study. In the literature, very different numbers were presented; ^4,13,16,18^ even in several studies using the same product.^26-28^More recently, Cohen et al^29^ published a comparison of 2 commercial mechanical stromal cell isolation products. The first one was the Tulip NanoTransfer kit (Tulip Medical, Inc., San Diego, CA, USA), which consists of 2.4, 1.4, and 1.2 mm connectors and 400 and 600 microns 2-layered filters. The other one was the LipocubeNano (Lipocube Inc., London, UK) device that consisted of 4 ports and a 500-micron single filter. They found that LipocubeNano produced a cell count of 2,240,000 cells per mL and cell viability of 96.75%, and Tulip’s NanoTransfer method resulted in 1,440,000 cells per mL with a cell viability of 96.05%. Both systems perform mechanical separation with blunt-edge filters, not a sharp blade system. However, in Adinizer, and in both of our techniques, the main philosophy is to avoid excessive blunt pressure and cut the fat with sharp blades. Adinizer plays an important role in the sharp blade edges (Figure 1) and protrusions of through-holes and is the biggest difference from Lipocube and other products.

Various methods are used to evaluate the number and viability of the stromal cells obtained. These include counting chambers/hemocytometers, automated cell counters, Coulter counters, flow cytometry, and DNA weight analysis devices.^4,16,27^ Although the most widely accepted is flow cytometry, it is not practical to apply this device in operating room conditions. Automated cell counters are devices that give very practical and effective results that are generally developed for enzymatic methods. There are no studies in the literature about the results and reliability of these devices, especially in mechanically obtaining stromal cells. In this study, we compared dual fluoroscopy, a practical device, with flow cytometry for the first time. The results were very close between the two. Although better results are obtained in flow cytometry, we think that particle dual fluoroscopy devices can also be used as a guide for the surgeon.

In our study, for the first time in the literature, IPs were defined, and stromal cells were obtained in different cell numbers in different volumes for different indications. Differences between cell numbers can be explained by predilution with saline. Predilution leads to better cutting of fat tissue and better isolation of stromal cells. We speculate that these effects can be explained by not only the difference in density but also the interaction and polarity between adipose tissue/saline; further studies are needed to confirm this. However, with the predilution method, just as with enzymatic methods, stromal cells in solution form were obtained providing ease of application. In their study, Sesé et al^16^ proved that the main stromal cells are in the adipose tissue excreted in enzymatic methods. However, this fat tissue had to be discarded because it contained tissue enzymes. Since there is no foreign material used in our technique, this adipose tissue is not excreted. We believe that there are abundant stromal regenerative cells in adipose tissue that exist in IPs 3 and 4. For this reason, we observed much more skin-quality change in the areas where we applied the fat tissue remaining in IPs 3 and 4 to the face. Mechanical methods have many advantages over enzymatic methods.^14,15^ The enzymatic disruption of adipose tissue results in a single-cell suspension in which all cell–cell communications are fully disrupted, and the ECM is digested; adipocytes are destroyed too. After mechanical isolation, however, adipocytes are also destroyed, but intercellular connections and cell–ECM connections remain intact.^30^ Since sharp blades were used in our study, the structure of adipocytes is also preserved. Although we presented cell viability histopathologically with hematoxylin-eosin, that cells could be undergoing apoptosis, yet still maintain otherwise normal architecture.

Even after the last centrifuge, there is still intact adipose tissue, which is used for soft tissue augmentation. Moreover, the ECM, which is an important reservoir of growth factors and acts as an instructive scaffold in the regenerative processes, also remains intact, in contrast to enzymatically dissociated lipoaspirate. When adipose tissue is enzymatically digested, the architecture and instructive capacity of the stromal tissue are fully destroyed, although the isolated stromal tissue cells will survive.^30^ More effective stromal cells can be obtained by mechanical methods in greater numbers and with more compositions for wound healing and regeneration.^16^ Mechanical shear stress is always created in these techniques, which may lead to the upregulation of multipotent and pluripotent markers that connote regenerative capacity.^31^ Mechanical disaggregation requires 10 times less fat tissue as the starting material to provide a similar or even higher cell dose, compared with conventional enzymatic SVF isolation.^16^ In addition to enhanced cell yield performance, mechanically disrupted cell aggregates (nanofat) remain attached to their natural matrix niche, which has been shown to promote cell viability, proliferation, and differentiation.^16^ Moreover, obtaining SVF enzymatically is time-consuming and expensive, requiring equipment and personnel, and above all, it is an application that is classified as a biological drug by authorities, such as the US Food and Drug Administration and European Medicines Agency, meaning that conditions of cGMP and cGLP must be met, which is impossible for many hospitals and doctors.^12^

It is unclear how many stromal cells should be given to which tissue, but Sesé et al^16^ described the cell dose. IPs allow different approaches for both the desired number of cells and the desired end product form. For example, if the stromal cell will be applied to the face or hair with needling only in the solution form, IP 1 or 2 is preferred, depending on the amount of fluid required, while IP 3 or 4 is preferred if the fat will be used to increase tissue retention.

Coleman and Katzel^32^ described the structural fat grafting technique, which should be considered as the standard and preferred method for harvesting, processing, and placing fat in the face. Yoshimura highlights the secret of successful fat grafts in his chapter in Coleman’s book, which will be regarded as the bible of lipofilling practices.^32^ There are 2 ways to enhance the relative number of stem cells in the graft—reducing the number of adipocytes and tissue volume or increasing the number of ASCs. Increasing the number of ASCs can be done by supplementing freshly isolated SVF or cultured/purified ASCs to the graft (cell-assisted lipotransfer).^33^ In the ARAT method we defined, we believe that we actually achieve “simultaneous cell-enriched lipotransfer” because we unbind parenchymal and stromal cells with sharp blades. There are 2 main complications in fat grafting—visibility in thin skin and low retention rate. Applying different size adipose tissue grafts to different anatomic areas will prevent the visibility of fat tissue. We speculate that the separated stromal cells actively lead to a better fat graft application and higher retention rate, but further studies are required to verify this speculation. Another limitation of this study is that cases were evaluated with VAS. Although visual outcomes are helpful, they merely support general safety and satisfaction and are not objective ways to differentiate one technique from another.

Unlike other techniques, since we cut the fat tissue with a sharp blade-edge system, not a blunt-edge filter system, we do not create an excessive blunt pressure, and without killing adipose tissue, in the same session, both the desired size and the fat graft can be prepared; in many ways, superior stromal cells can be obtained than with enzymatic methods.

## CONCLUSIONS

With this study, a new approach has been introduced in which the size of the fat graft desired to be applied is defined numerically, and it has been allowed to determine the measurable fat graft instead of the mini/micro/nano. The death of parenchymal cells, adipocytes, exposed to excessive blunt pressure in classical nanofat applications was avoided with sharp blades, allowing adipocytes to remain intact and successful fat grafting. At the same time, stromal cells were successfully separated mechanically, affording greater viability in a higher number of cells.

By employing a new protocol for stromal cells obtained mechanically, a liquid SCS was obtained as with enzymes, and different protocols were applied for different indications. Finally, in the production of stromal cells by mechanical means, confusion was avoided by using TOST cell identification instead of SVF on the final product.

In combined applications, despite greater patient and surgeon satisfaction subjectively than with ARAT alone, the persistence of oil and regenerative effects and the permanence rate of fat tissue should be evaluated by objective imaging methods; the effect of MEST remains to be demonstrated mathematically. Although, we think that these results show that more permanent, more effective, and more successful fat grafting and facial regeneration can be done with ARAT and MEST techniques, they need to be proven by further studies.

## Supplementary Material

ojaa035_suppl_Supplementary_Figure_1A

ojaa035_suppl_Supplementary_Figure_1B

ojaa035_suppl_Supplementary_Figure_1C

ojaa035_suppl_Supplementary_Figure_1D

ojaa035_suppl_Supplementary_Figure_1E

ojaa035_suppl_Supplementary_Figure_1F

ojaa035_suppl_Supplementary_Figure_1G

ojaa035_suppl_Supplementary_Figure_1H

ojaa035_suppl_Supplementary_Figure_2A

ojaa035_suppl_Supplementary_Figure_2B

ojaa035_suppl_Supplementary_Figure_2C

ojaa035_suppl_Supplementary_Figure_2D

ojaa035_suppl_Supplementary_Figure_2E

ojaa035_suppl_Supplementary_Figure_2F

ojaa035_suppl_Supplementary_Figure_2G

ojaa035_suppl_Supplementary_Figure_2H

ojaa035_suppl_Supplementary_Figure_3A

ojaa035_suppl_Supplementary_Figure_3B

ojaa035_suppl_Supplementary_Figure_3C

ojaa035_suppl_Supplementary_Figure_3D

ojaa035_suppl_Supplementary_Figure_3E

ojaa035_suppl_Supplementary_Figure_3F

ojaa035_suppl_Supplementary_Figure_3G

ojaa035_suppl_Supplementary_Figure_3H

ojaa035_suppl_Supplementary_Figure_Legend

## Notes

This abstract was accepted for presentation at The Aesthetic Meeting 2020.
