# Supplementary figures and images for: New Mechanical Fat Separation Technique: Adjustable Regenerative Adipose-tissue Transfer (ARAT) and Mechanical Stromal Cell Transfer (MEST)
Source: Aesthet Surg J Open Forum. 2020 Jul 22;2(4):ojaa035. doi: 10.1093/asjof/ojaa035 (PMC7780457; doi:10.1093/asjof/ojaa035)

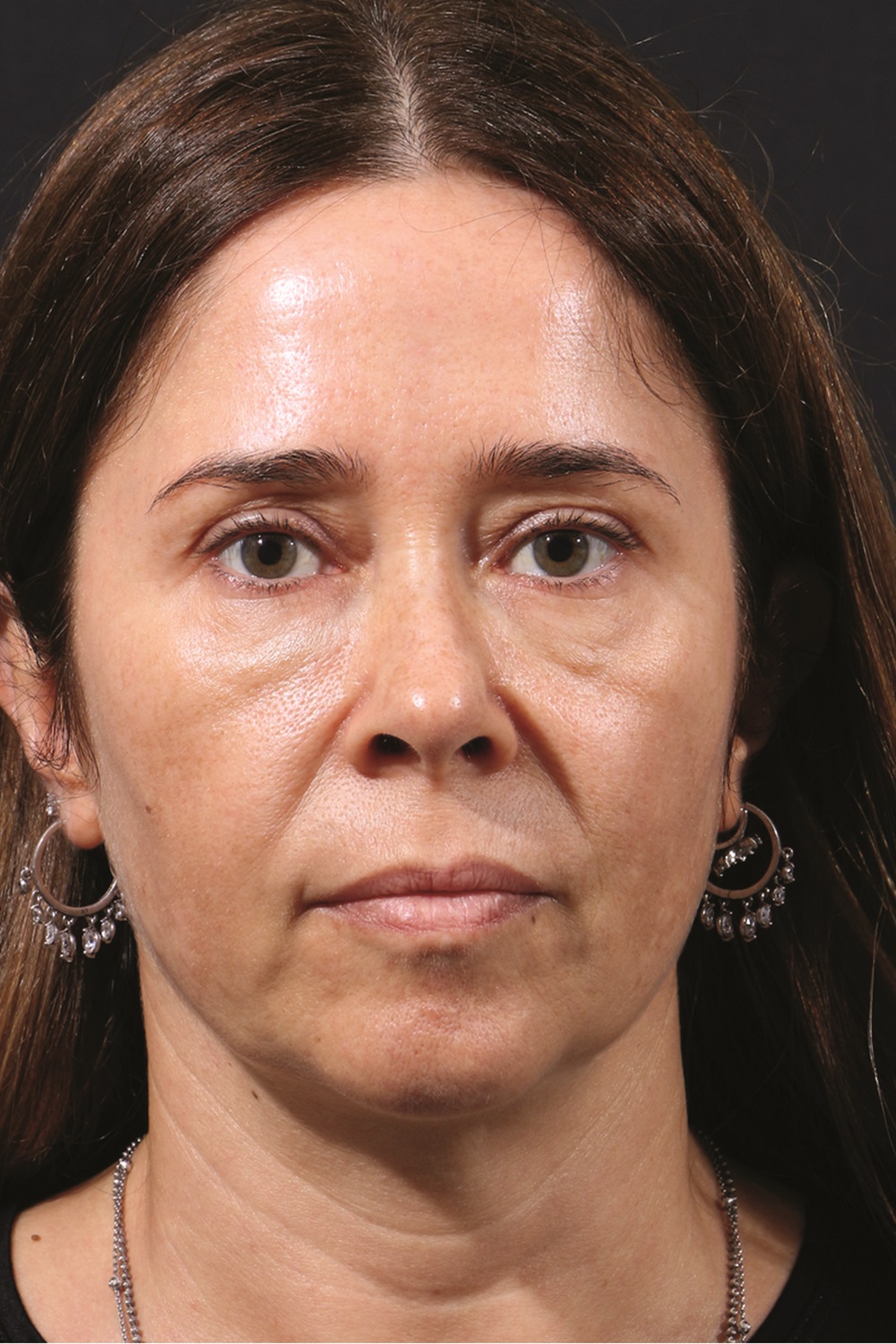

Supplement: ojaa035_suppl_Supplementary_Figure_1A [file ojaa035_suppl_Supplementary_Figure_1A.jpg]

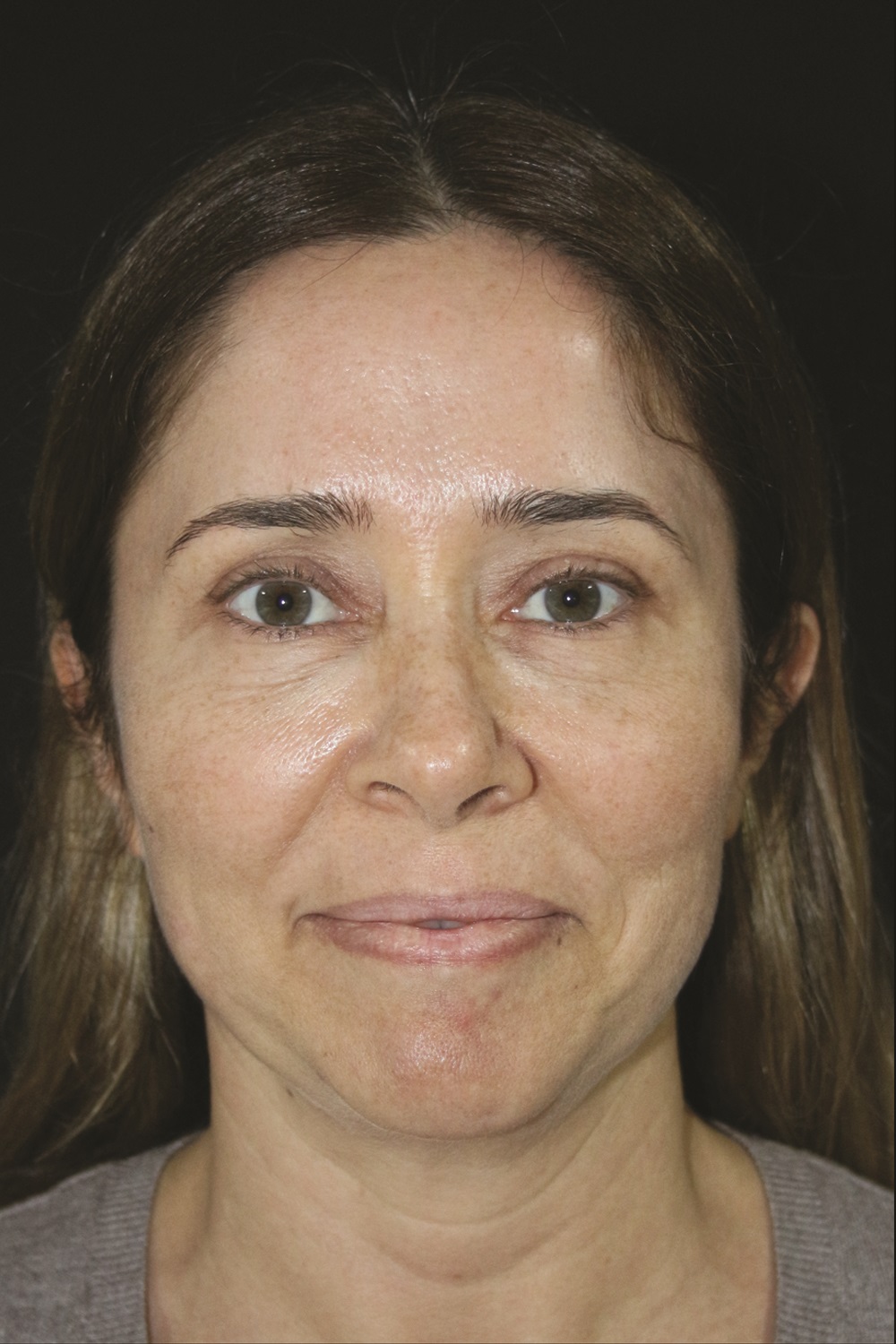

Supplement: ojaa035_suppl_Supplementary_Figure_1B [file ojaa035_suppl_Supplementary_Figure_1B.jpg]

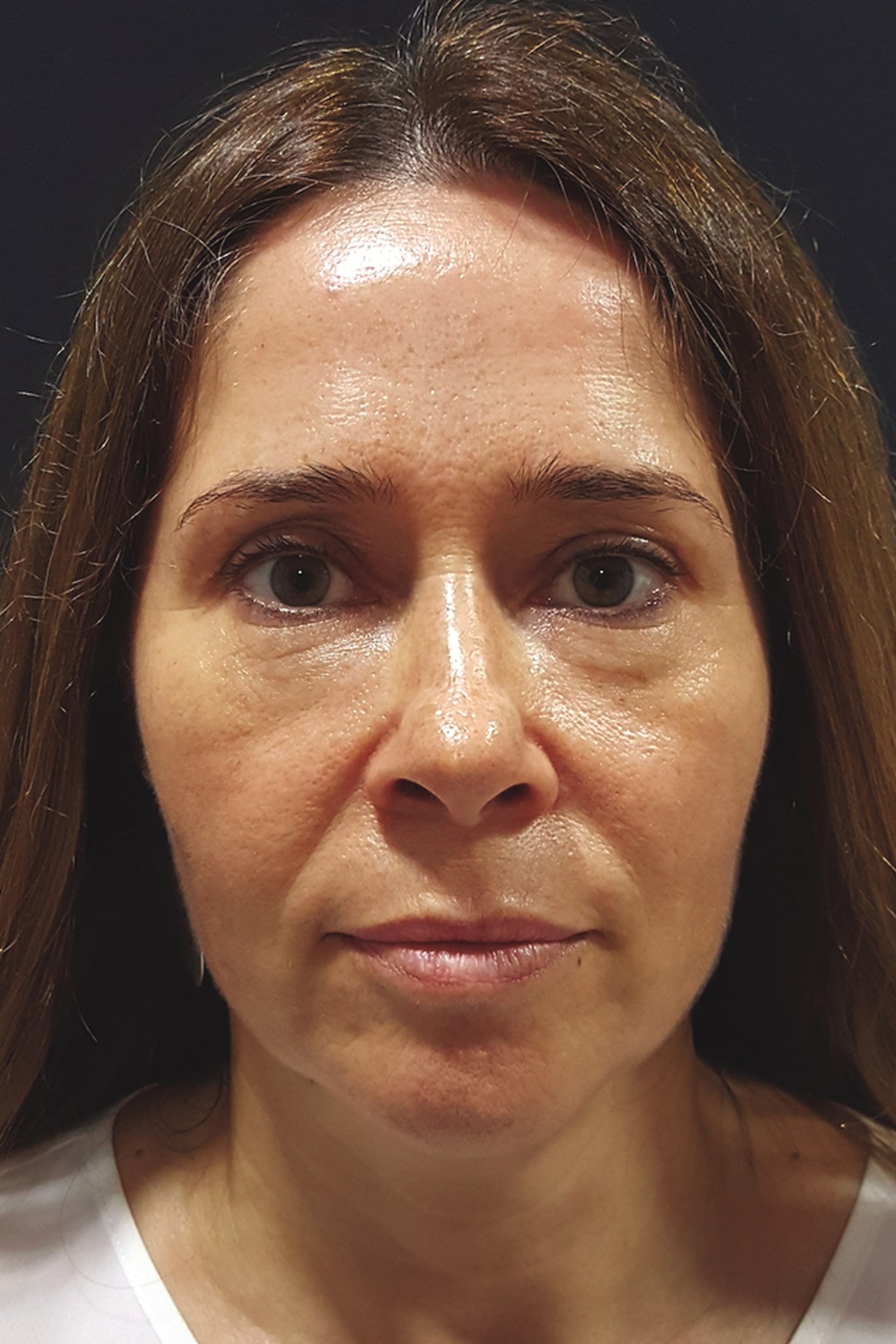

Supplement: ojaa035_suppl_Supplementary_Figure_1C [file ojaa035_suppl_Supplementary_Figure_1C.jpg]

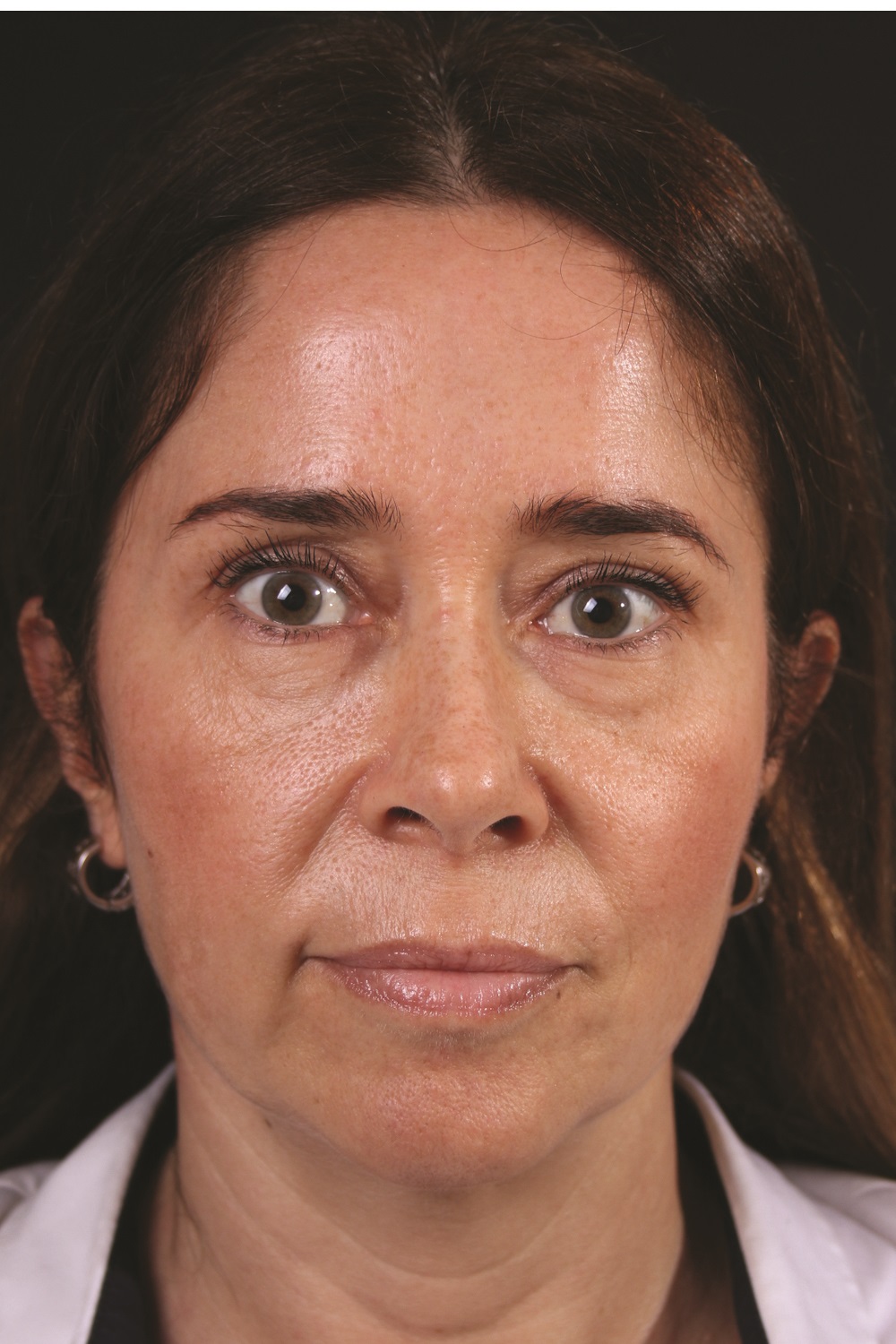

Supplement: ojaa035_suppl_Supplementary_Figure_1D [file ojaa035_suppl_Supplementary_Figure_1D.jpg]

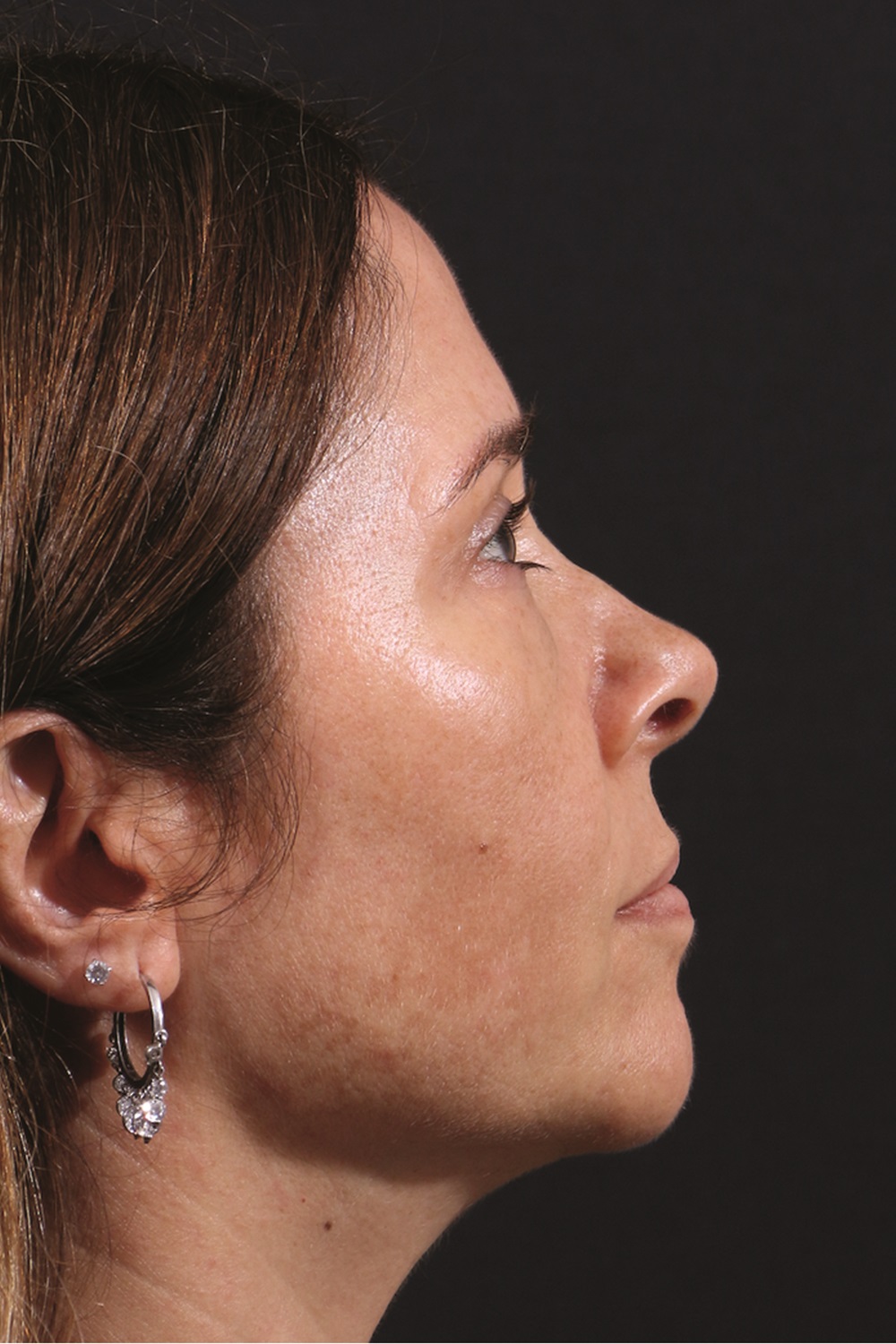

Supplement: ojaa035_suppl_Supplementary_Figure_1E [file ojaa035_suppl_Supplementary_Figure_1E.jpg]

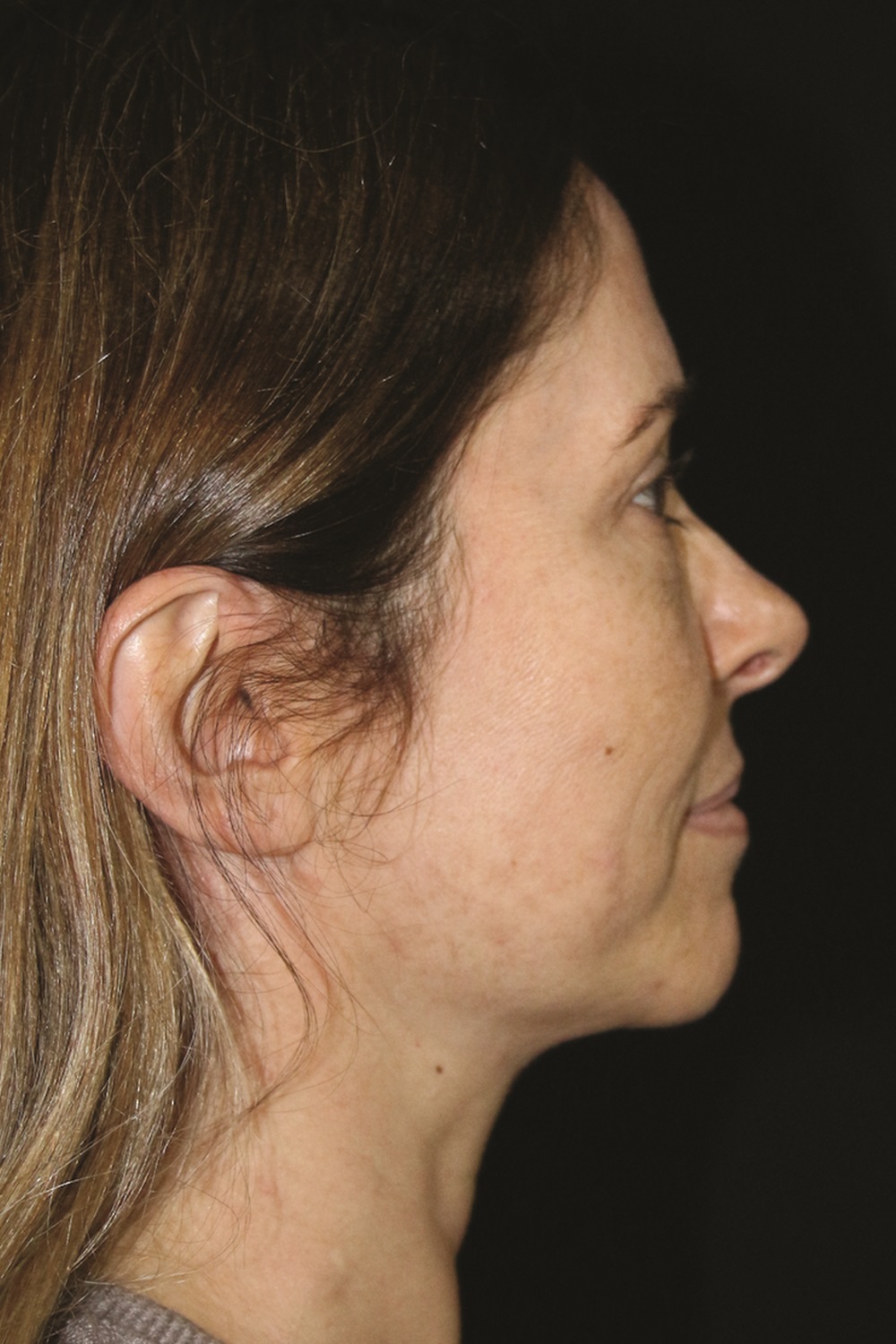

Supplement: ojaa035_suppl_Supplementary_Figure_1F [file ojaa035_suppl_Supplementary_Figure_1F.jpg]

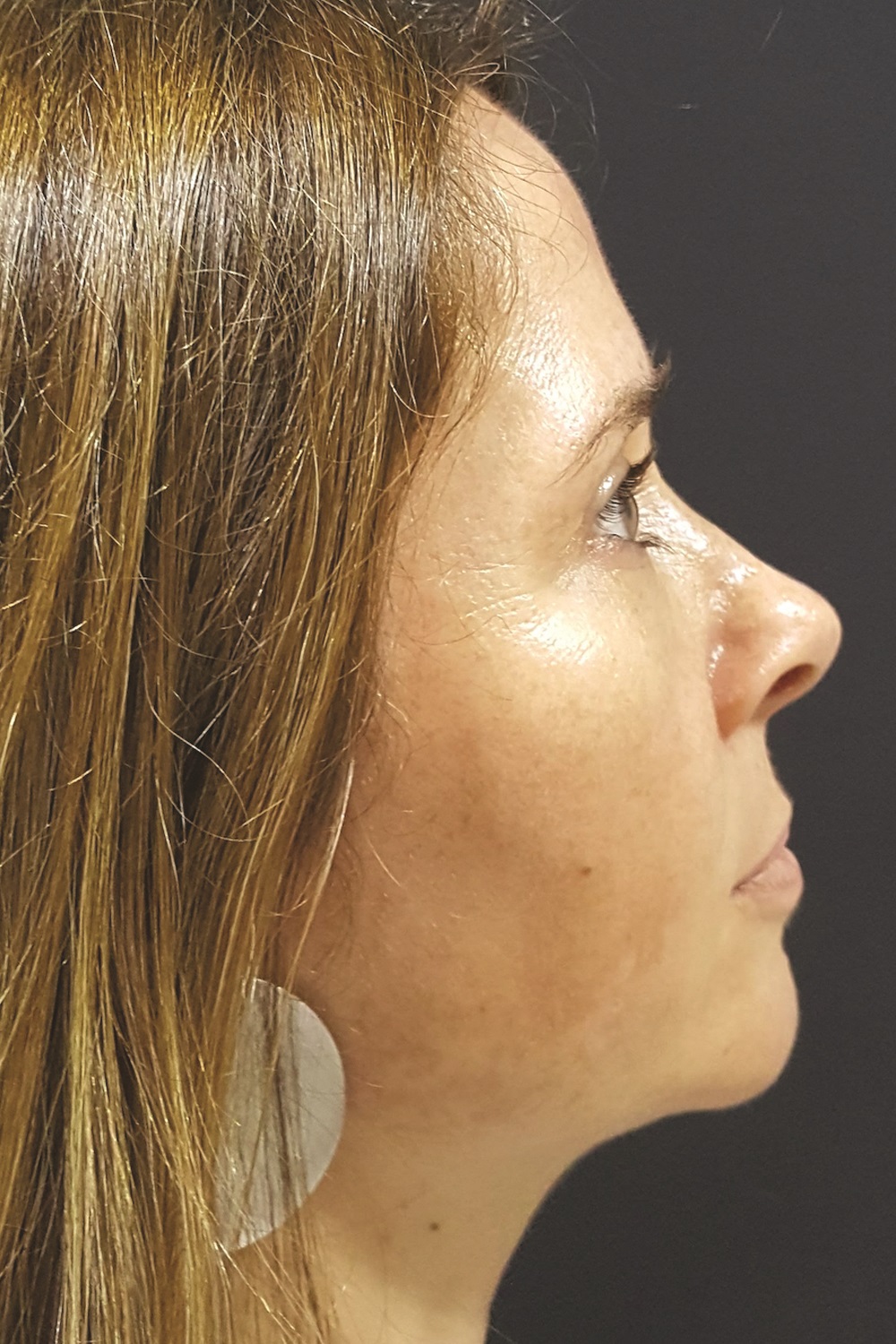

Supplement: ojaa035_suppl_Supplementary_Figure_1G [file ojaa035_suppl_Supplementary_Figure_1G.jpg]

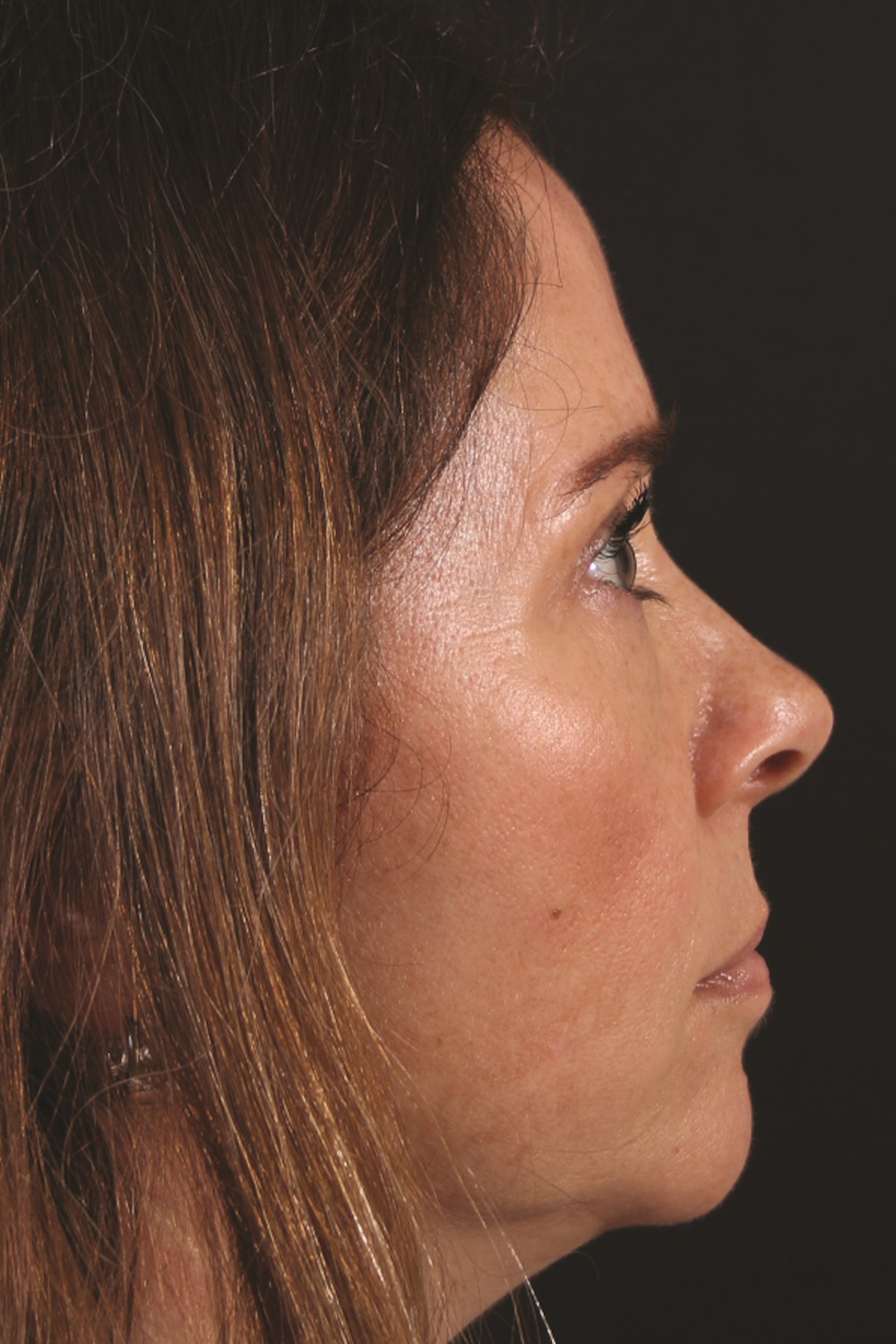

Supplement: ojaa035_suppl_Supplementary_Figure_1H [file ojaa035_suppl_Supplementary_Figure_1H.jpg]

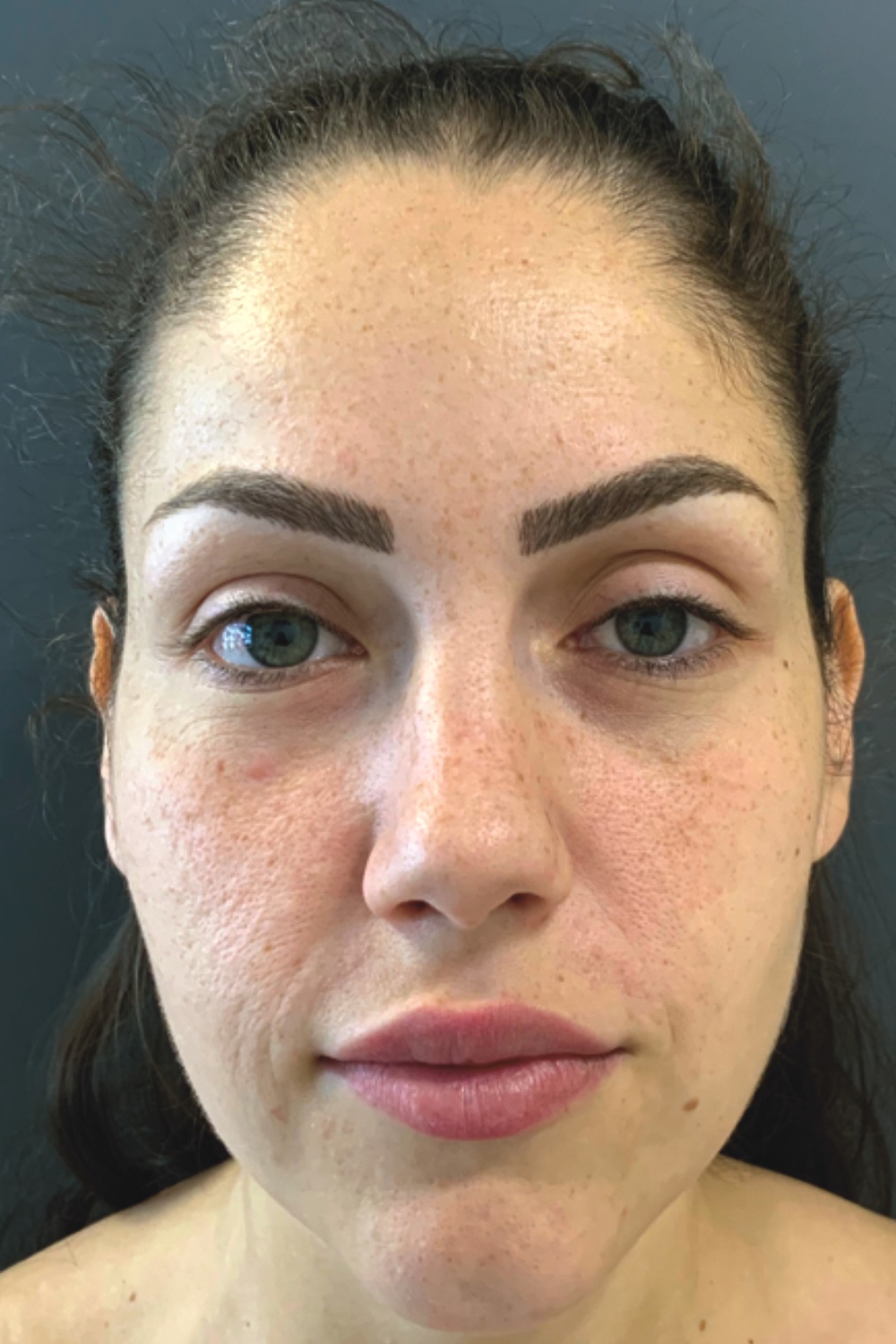

Supplement: ojaa035_suppl_Supplementary_Figure_2A [file ojaa035_suppl_Supplementary_Figure_2A.jpg]

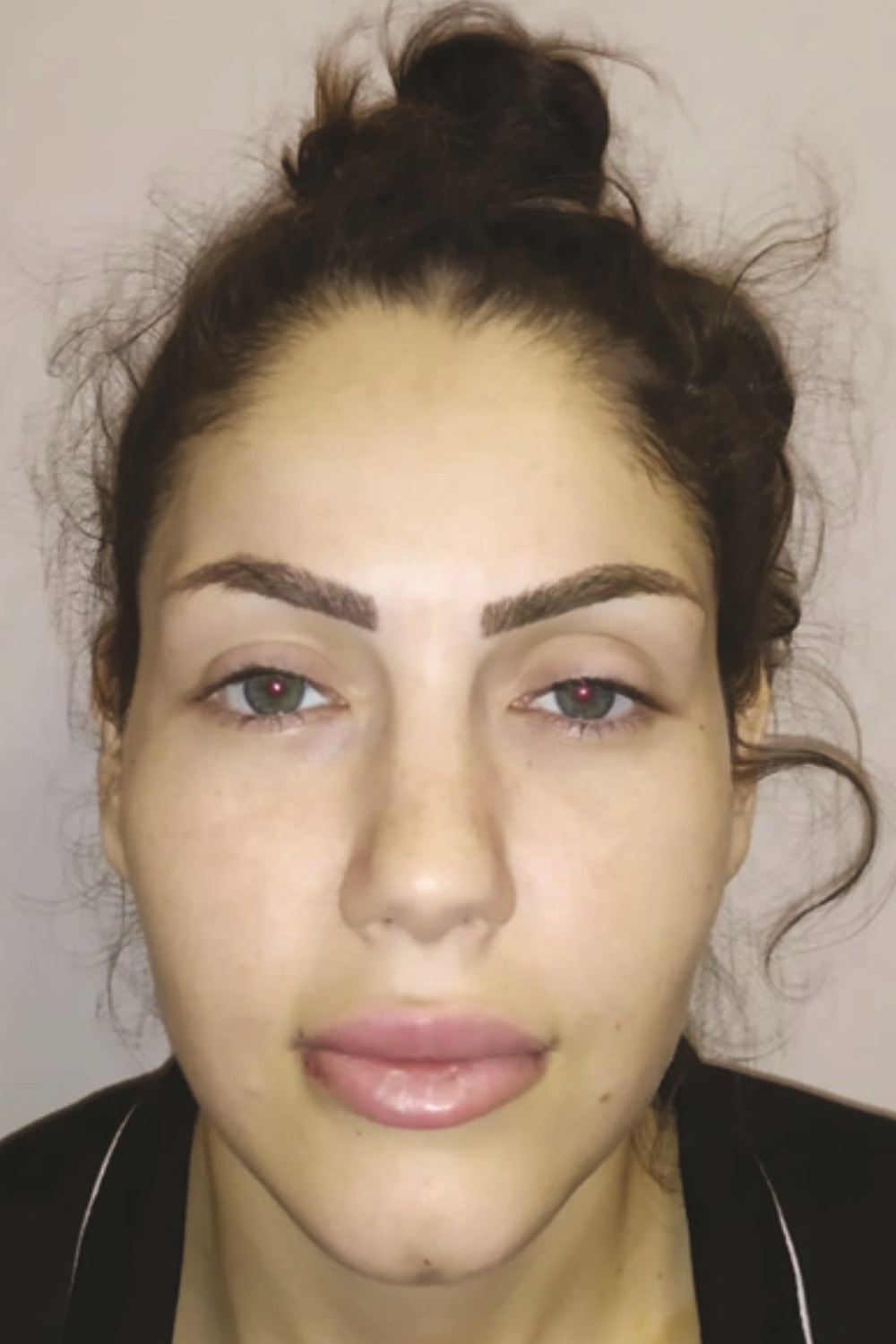

Supplement: ojaa035_suppl_Supplementary_Figure_2B [file ojaa035_suppl_Supplementary_Figure_2B.jpg]

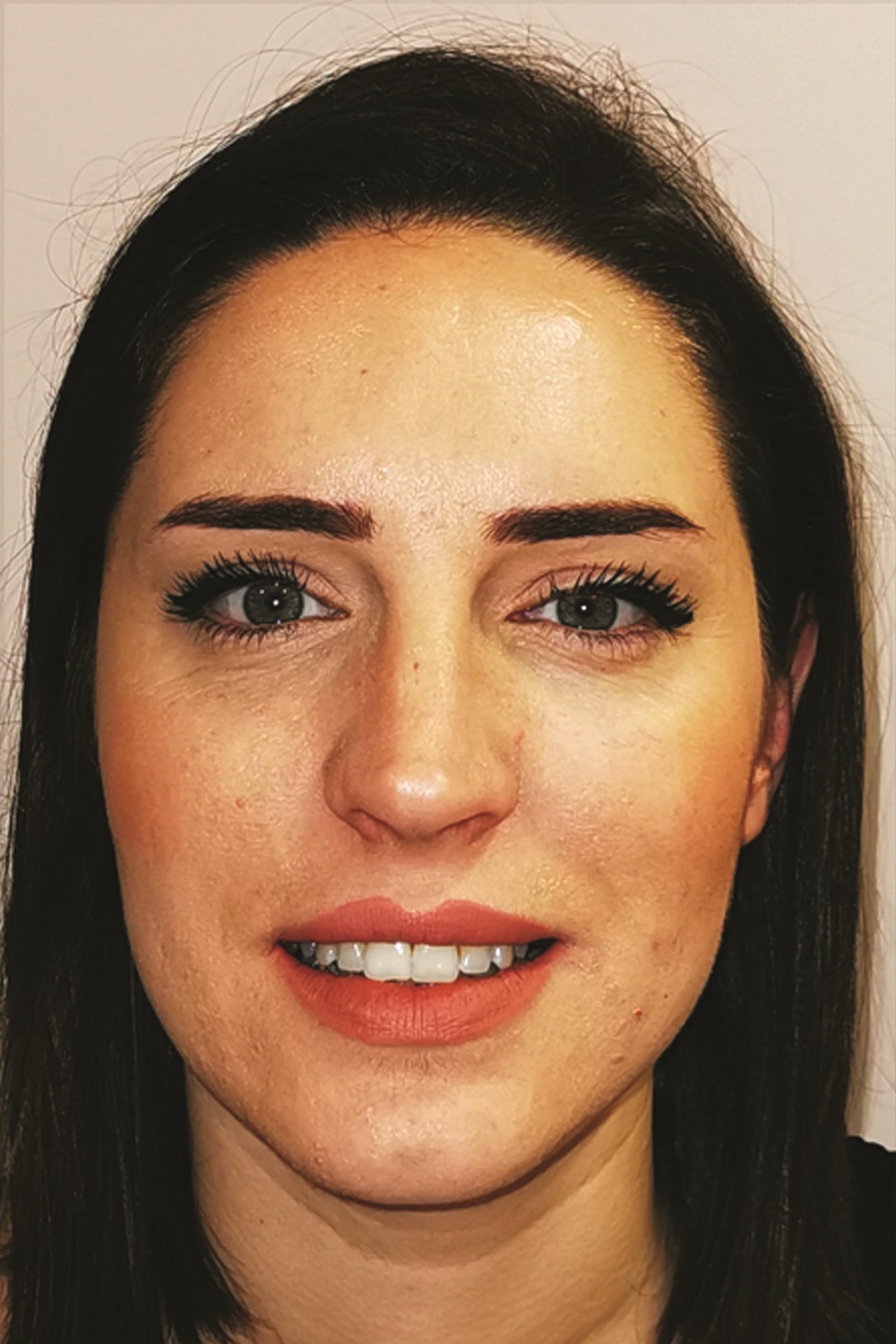

Supplement: ojaa035_suppl_Supplementary_Figure_2C [file ojaa035_suppl_Supplementary_Figure_2C.jpg]

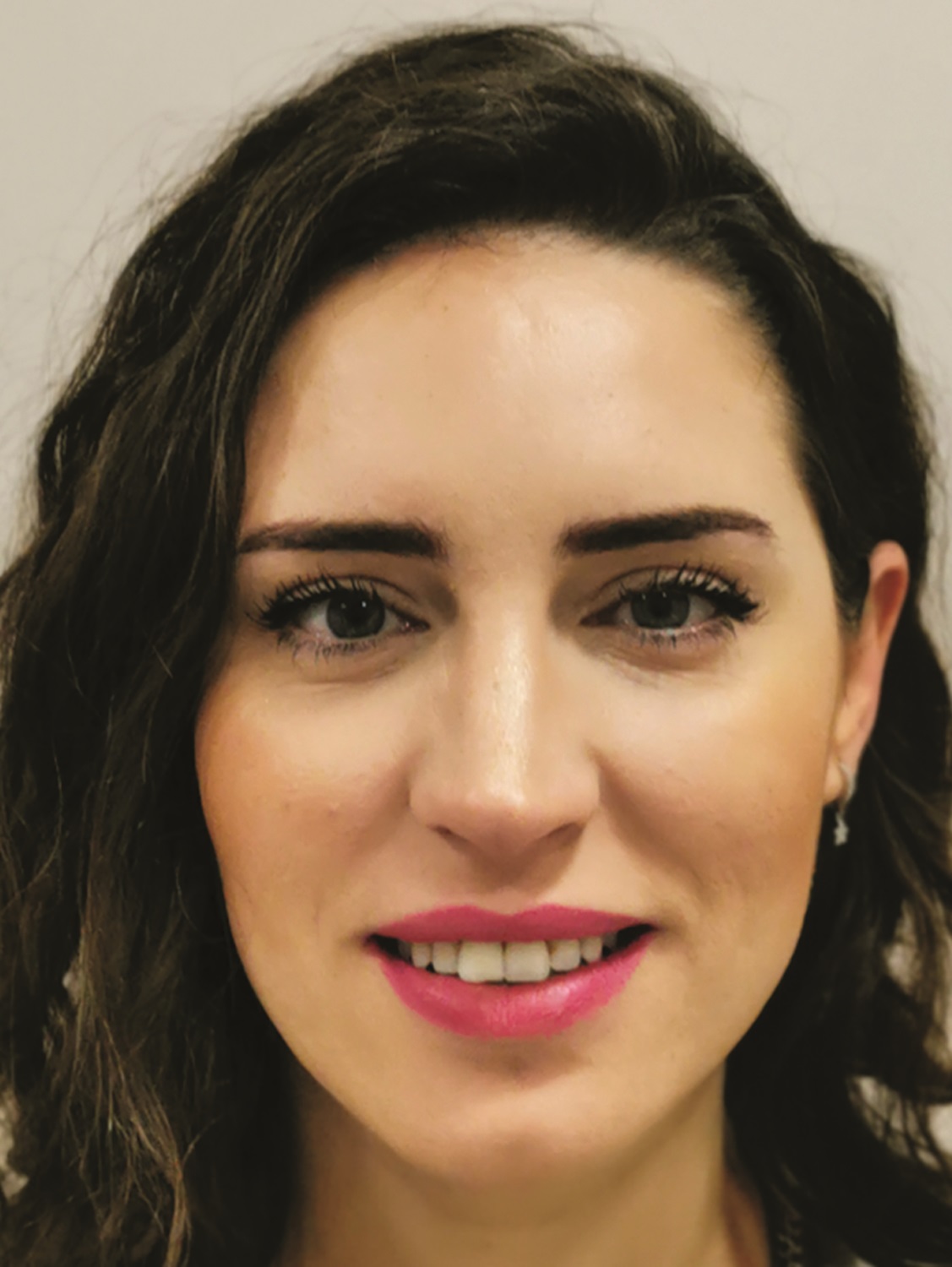

Supplement: ojaa035_suppl_Supplementary_Figure_2D [file ojaa035_suppl_Supplementary_Figure_2D.jpg]

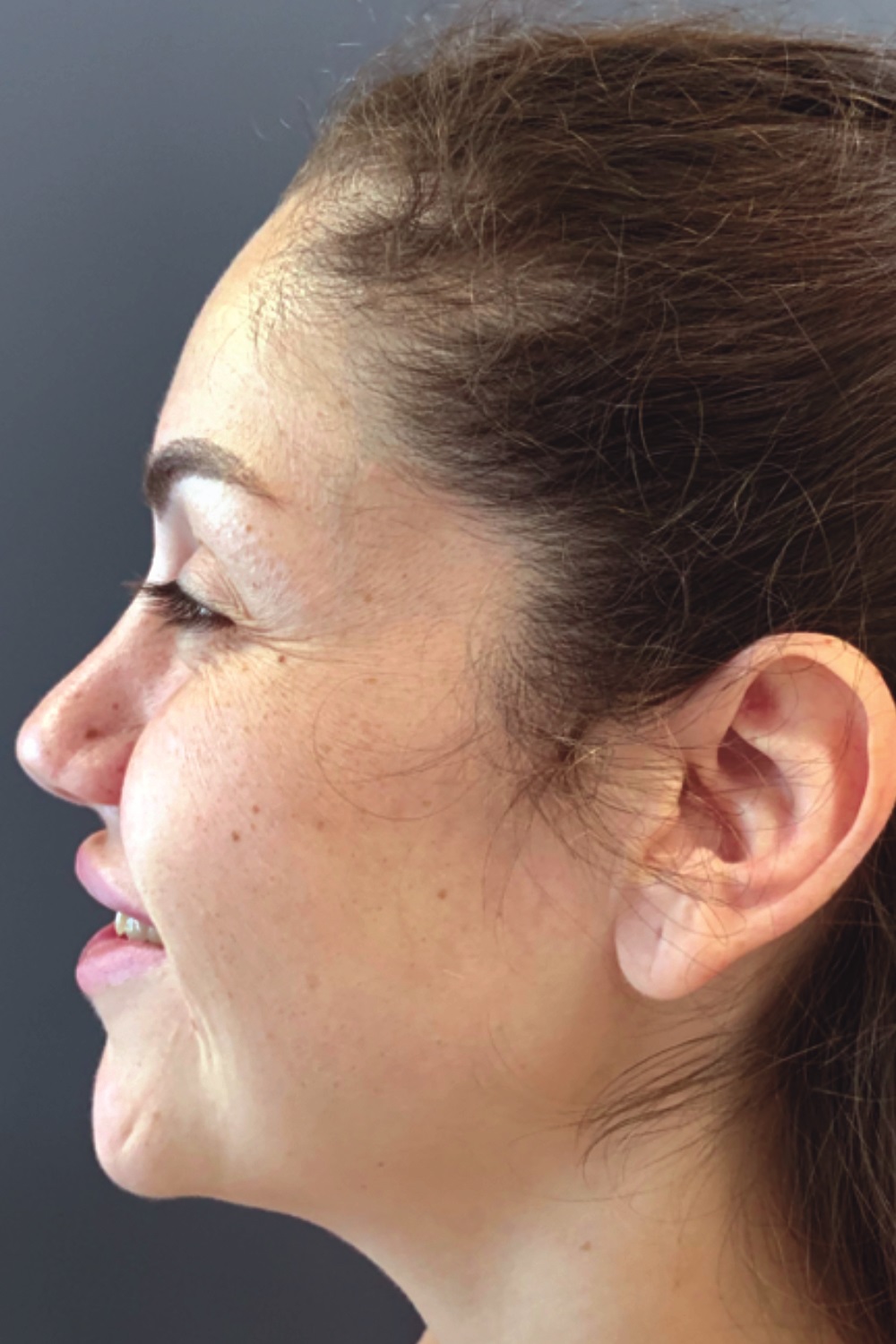

Supplement: ojaa035_suppl_Supplementary_Figure_2E [file ojaa035_suppl_Supplementary_Figure_2E.jpg]

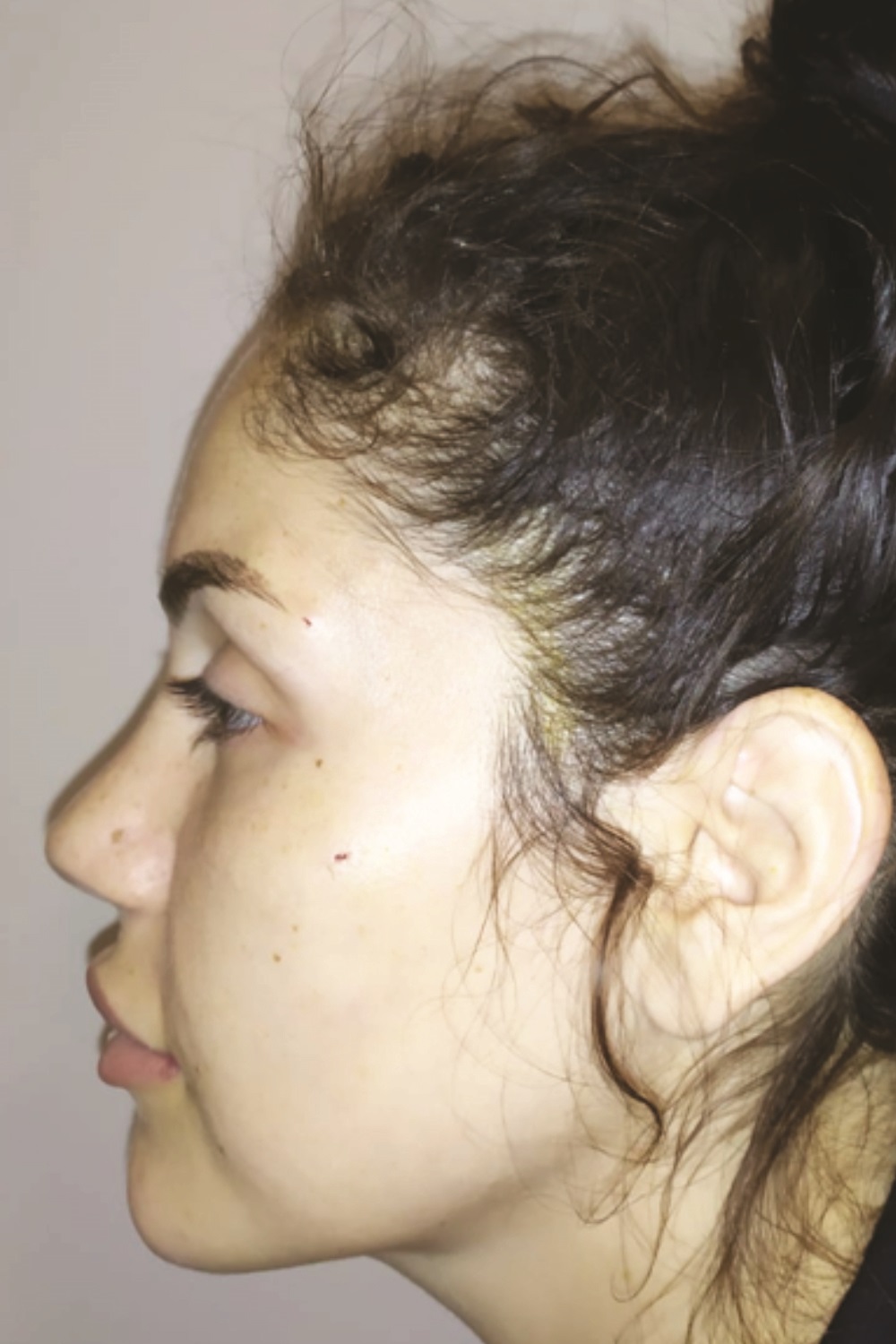

Supplement: ojaa035_suppl_Supplementary_Figure_2F [file ojaa035_suppl_Supplementary_Figure_2F.jpg]

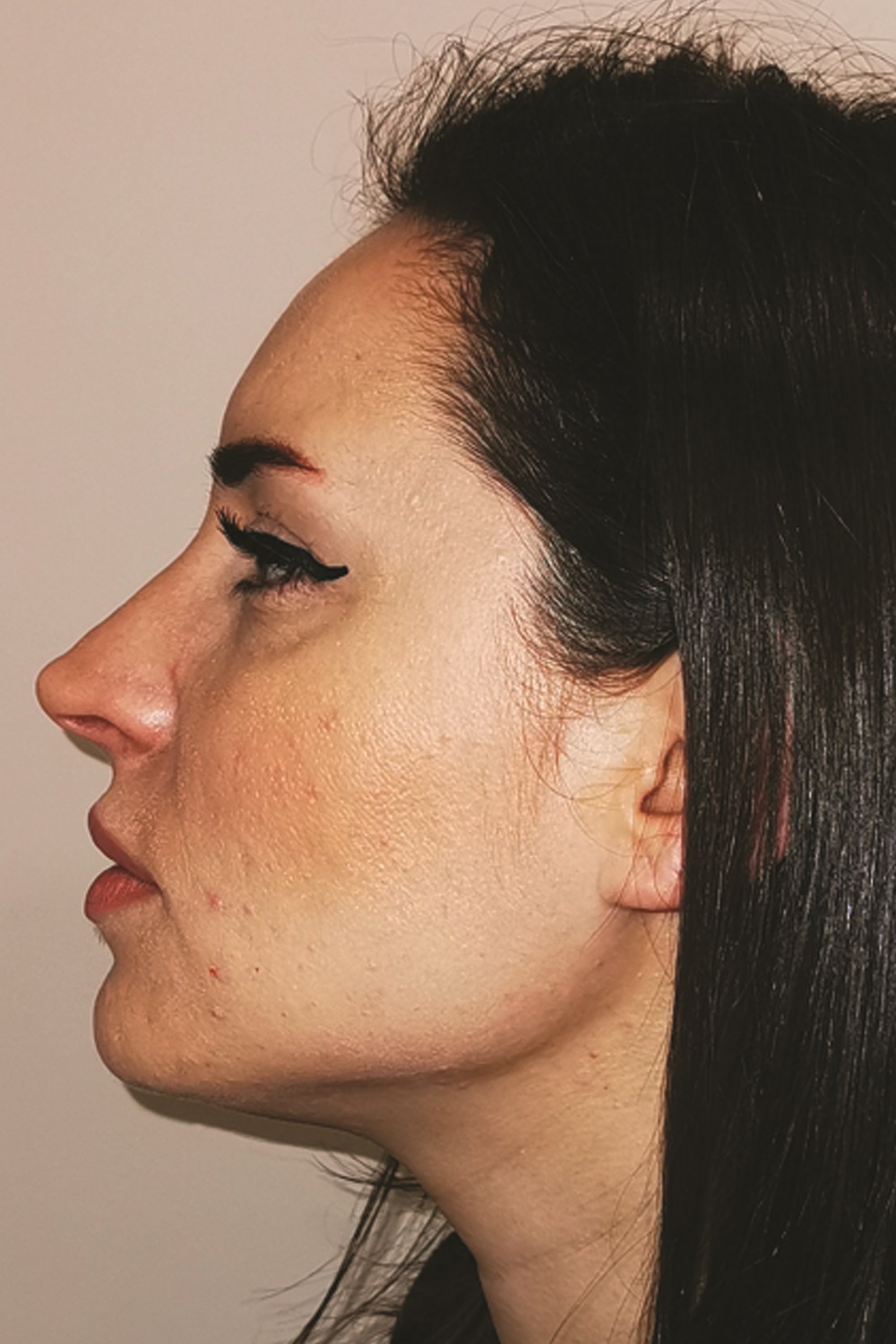

Supplement: ojaa035_suppl_Supplementary_Figure_2G [file ojaa035_suppl_Supplementary_Figure_2G.jpg]

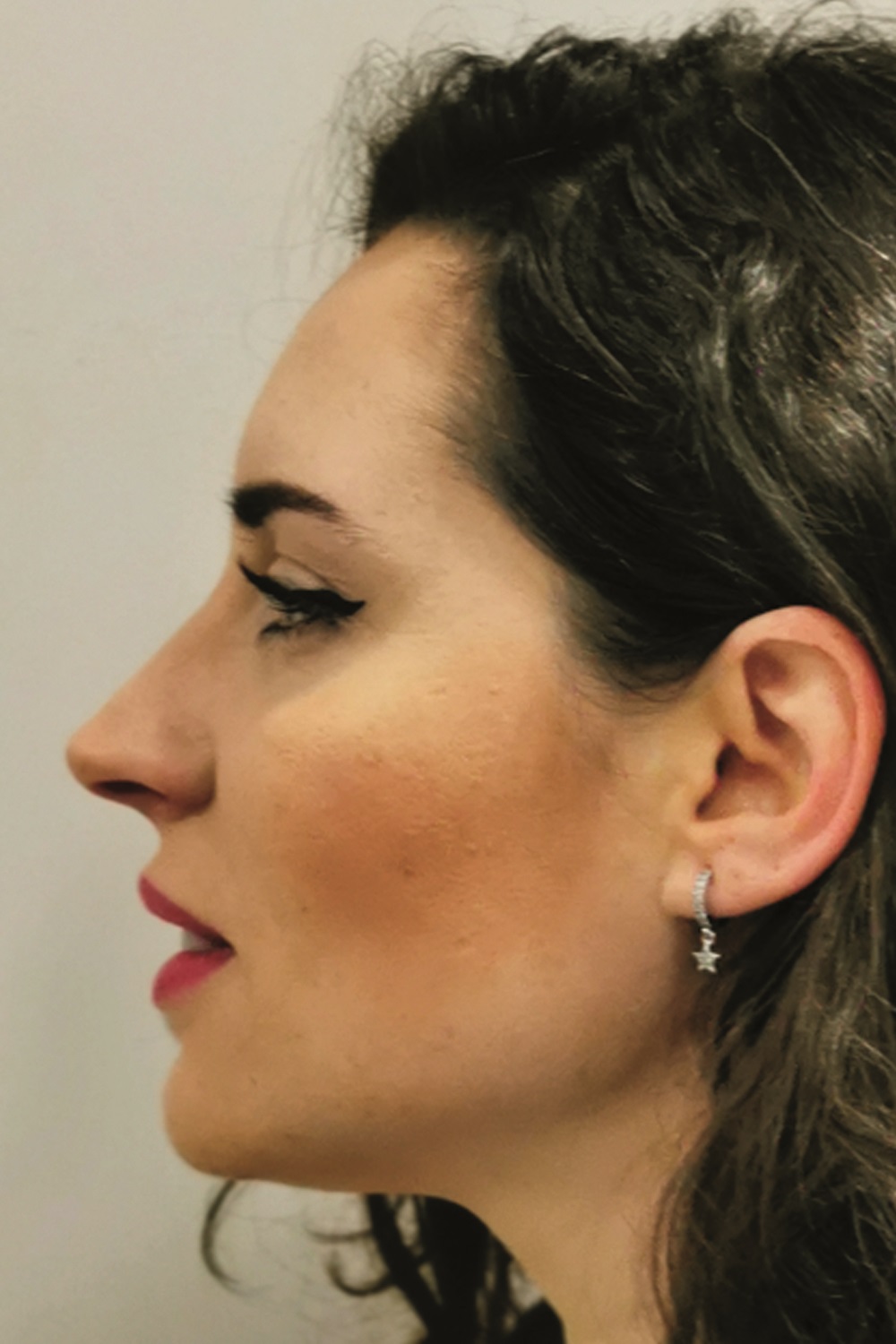

Supplement: ojaa035_suppl_Supplementary_Figure_2H [file ojaa035_suppl_Supplementary_Figure_2H.jpg]

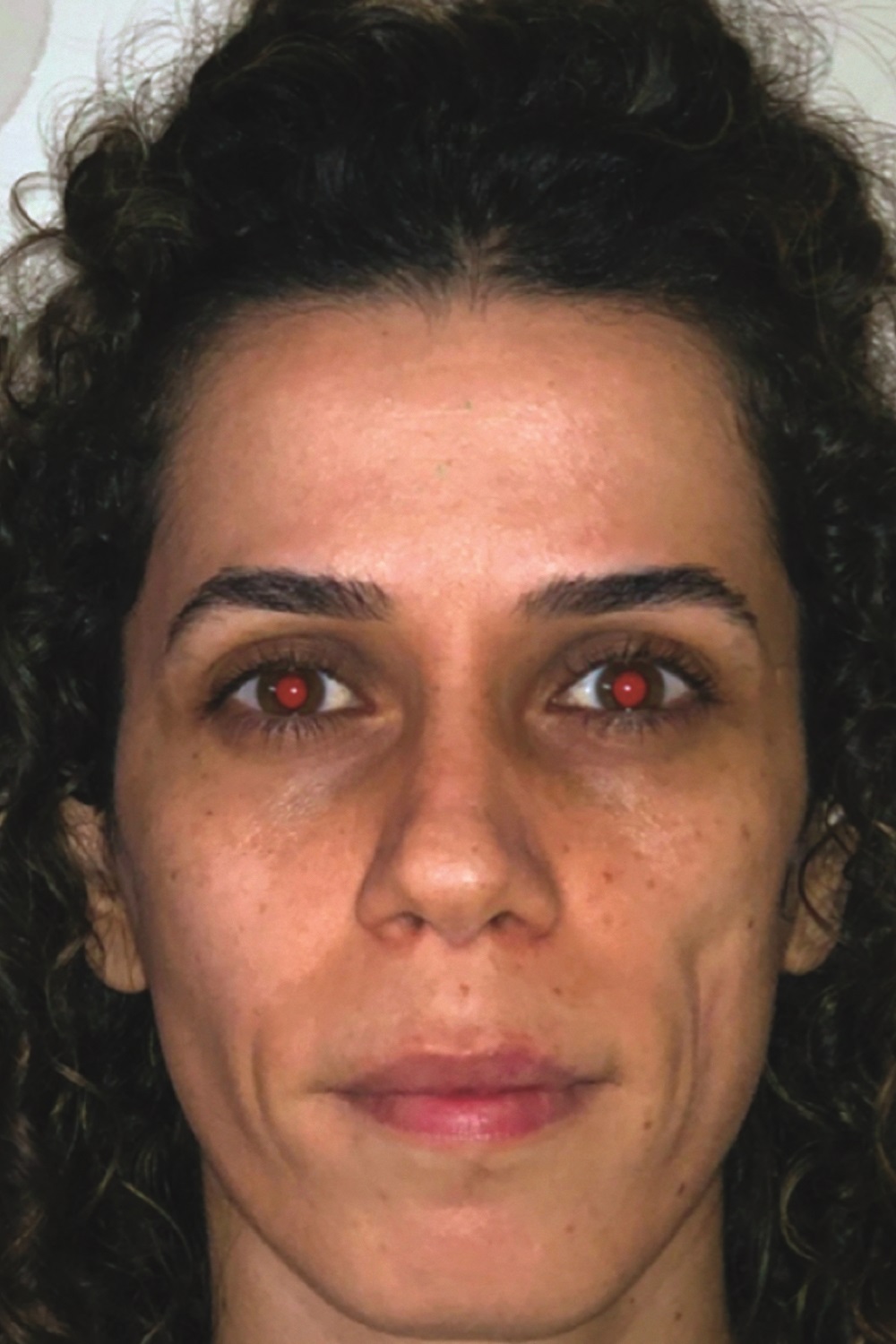

Supplement: ojaa035_suppl_Supplementary_Figure_3A [file ojaa035_suppl_Supplementary_Figure_3A.jpg]

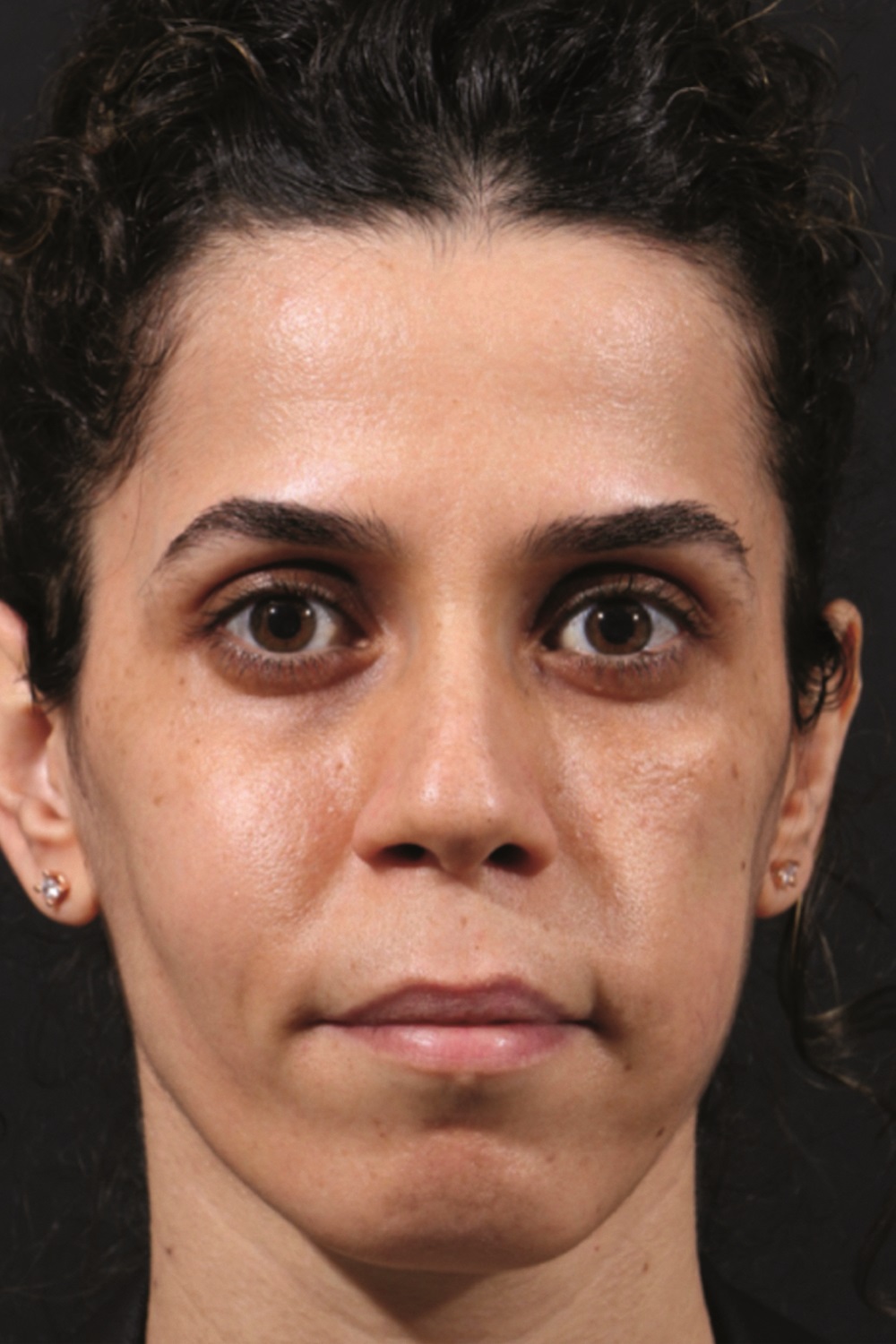

Supplement: ojaa035_suppl_Supplementary_Figure_3B [file ojaa035_suppl_Supplementary_Figure_3B.jpg]

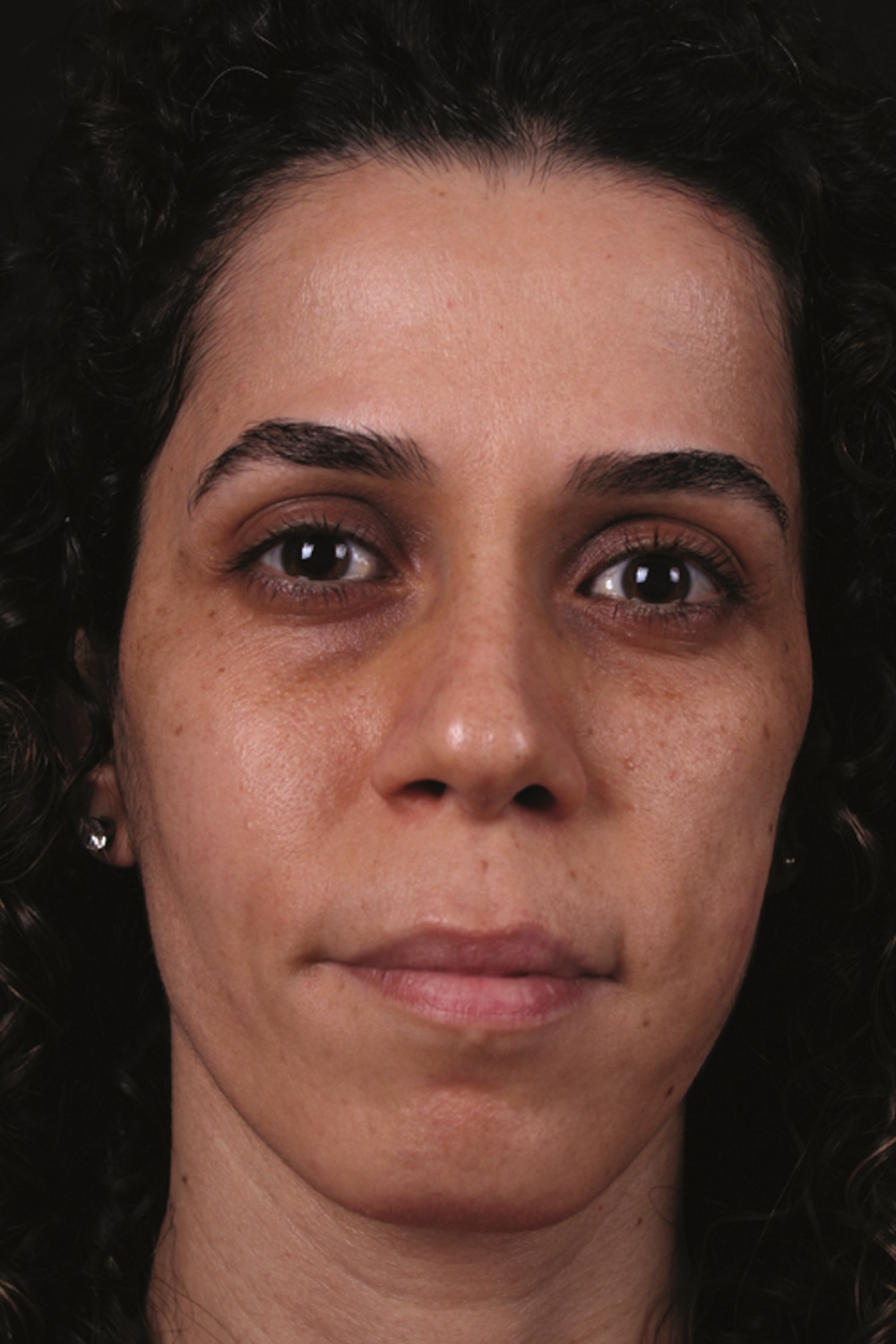

Supplement: ojaa035_suppl_Supplementary_Figure_3C [file ojaa035_suppl_Supplementary_Figure_3C.jpg]

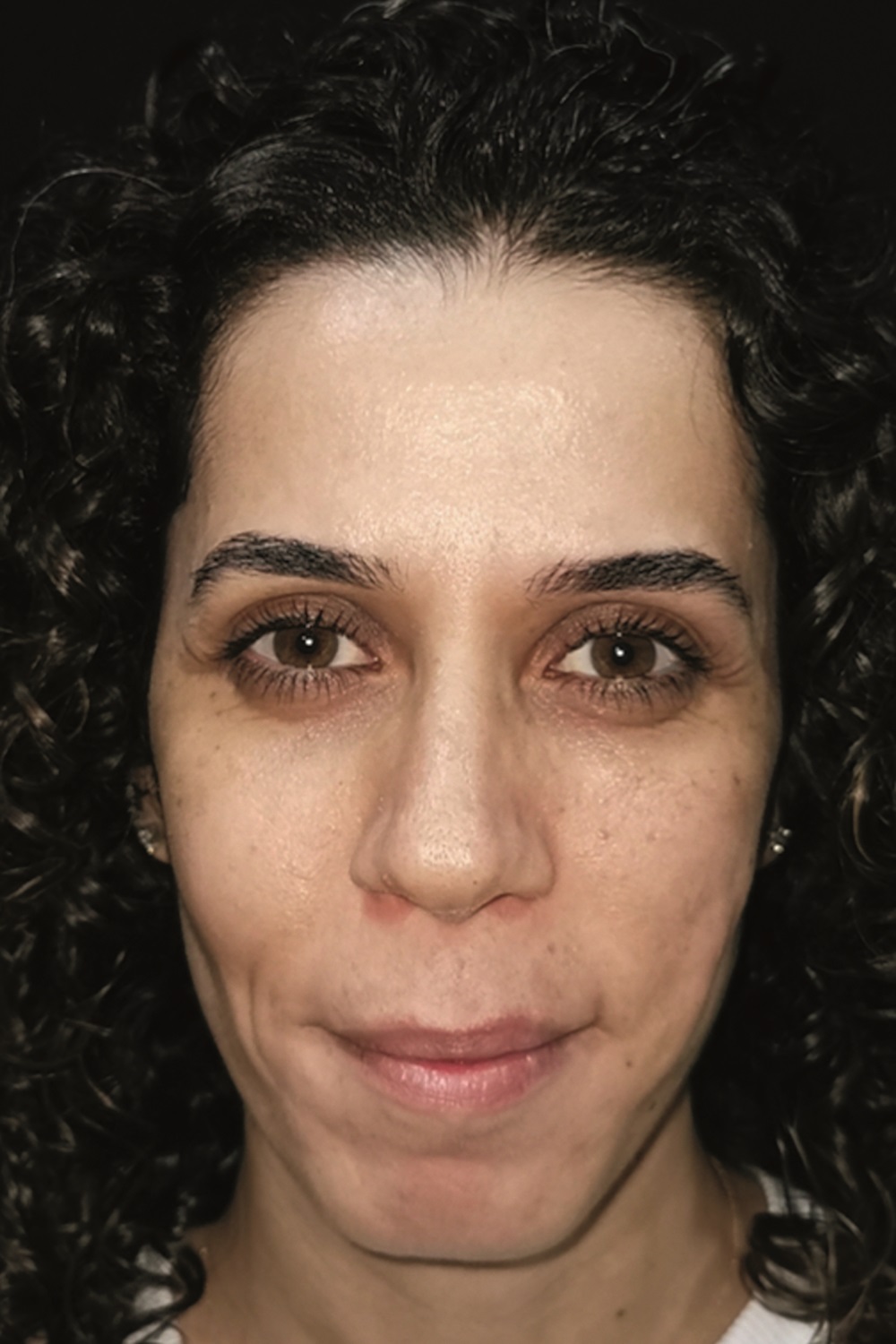

Supplement: ojaa035_suppl_Supplementary_Figure_3D [file ojaa035_suppl_Supplementary_Figure_3D.jpg]

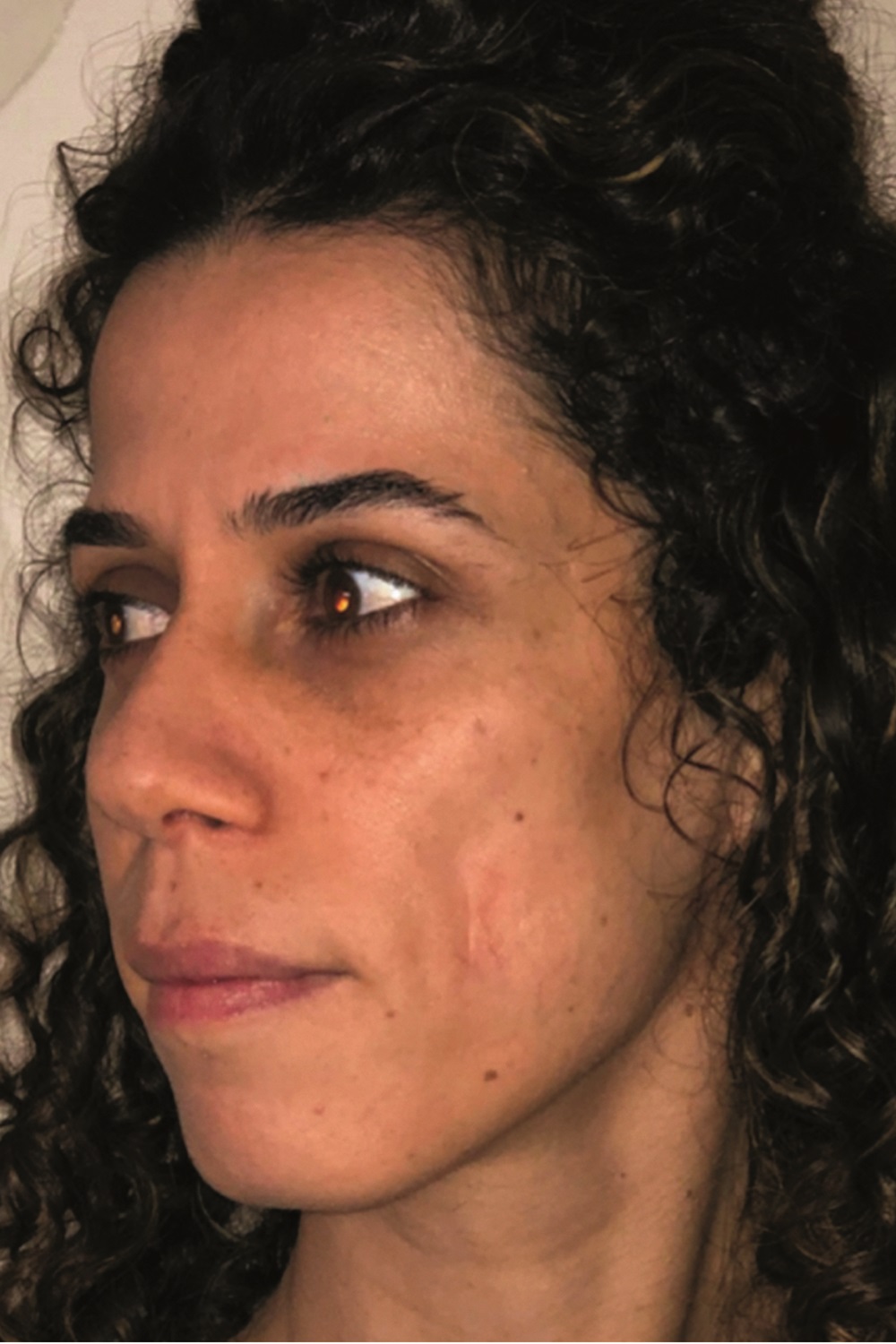

Supplement: ojaa035_suppl_Supplementary_Figure_3E [file ojaa035_suppl_Supplementary_Figure_3E.jpg]

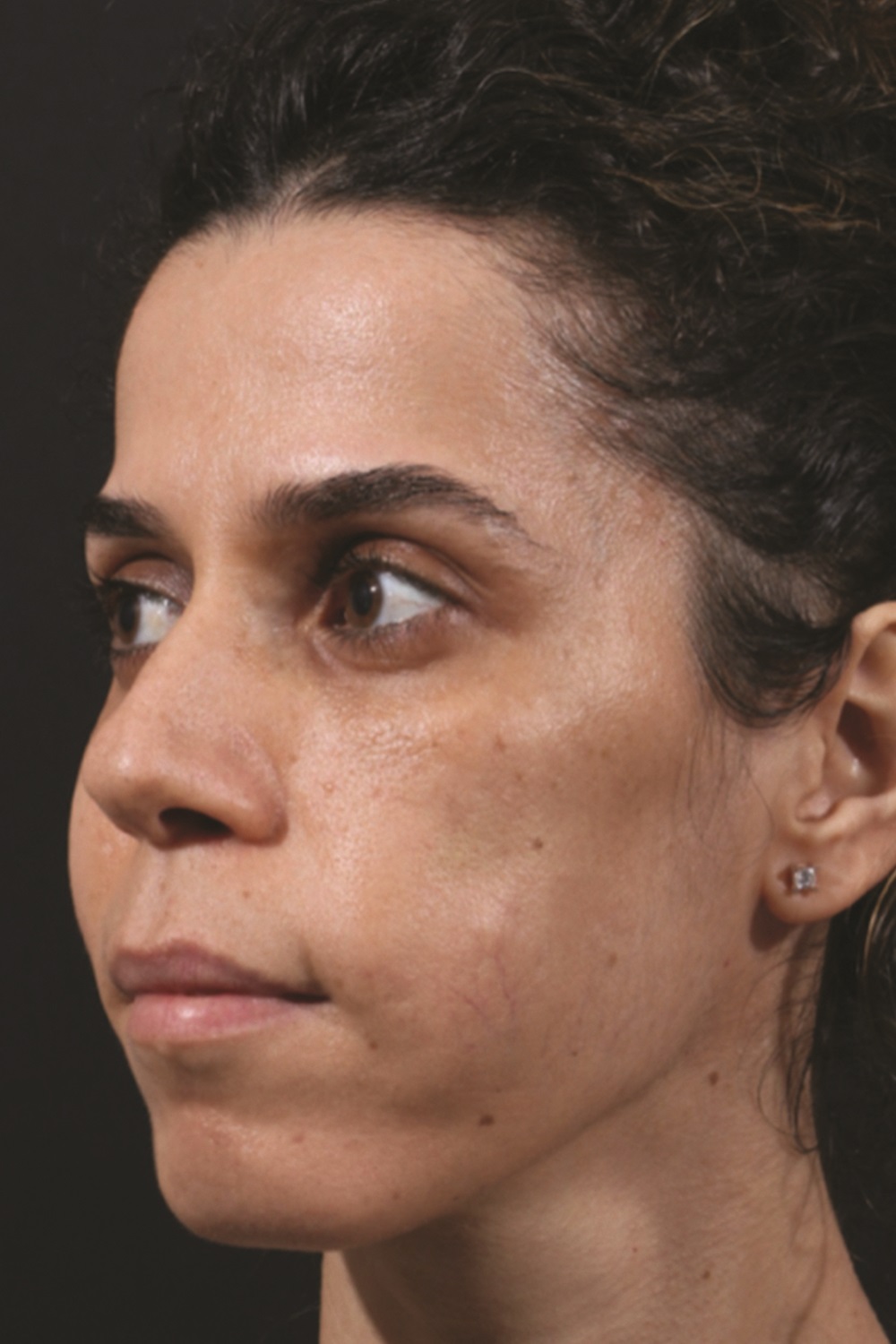

Supplement: ojaa035_suppl_Supplementary_Figure_3F [file ojaa035_suppl_Supplementary_Figure_3F.jpg]

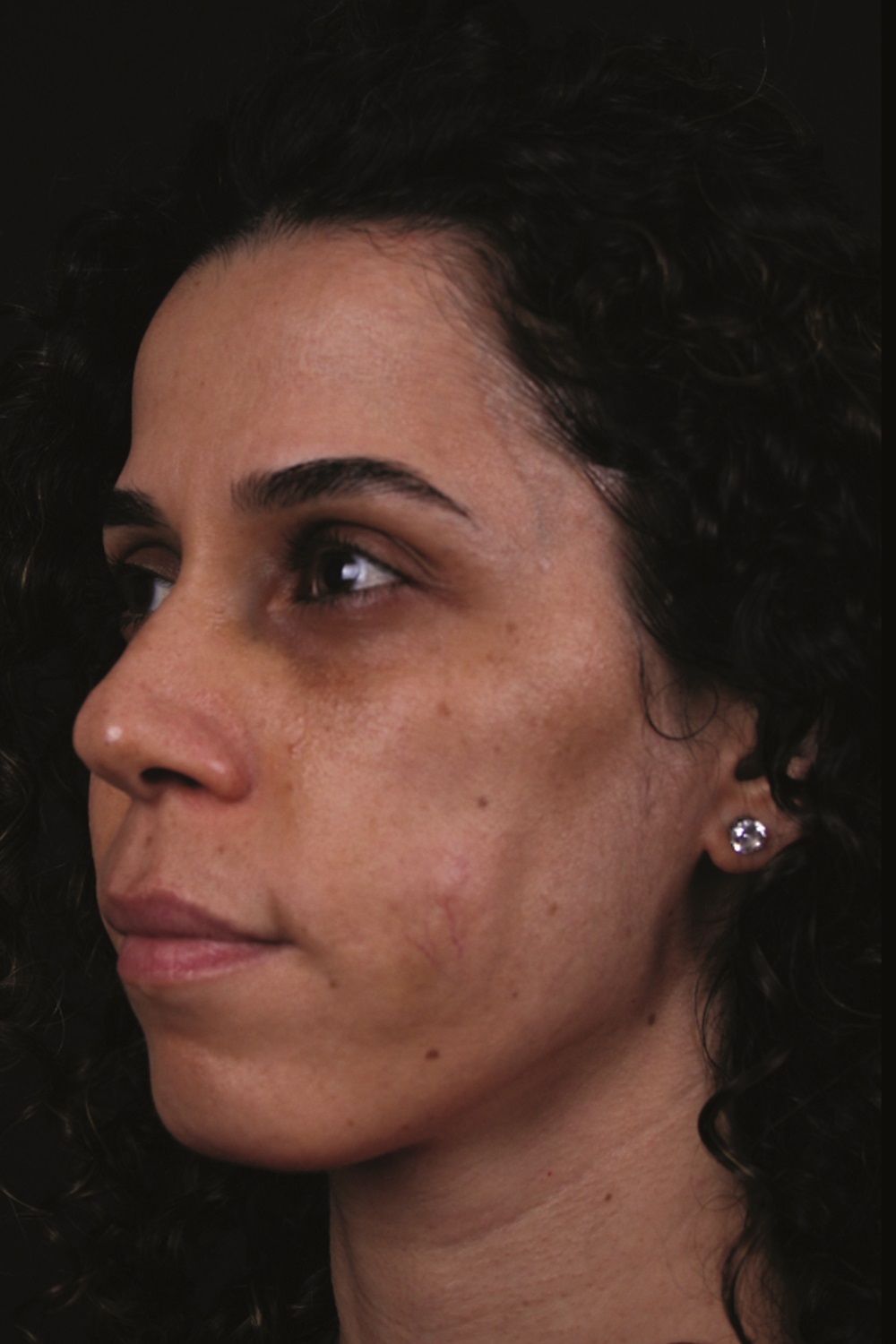

Supplement: ojaa035_suppl_Supplementary_Figure_3G [file ojaa035_suppl_Supplementary_Figure_3G.jpg]

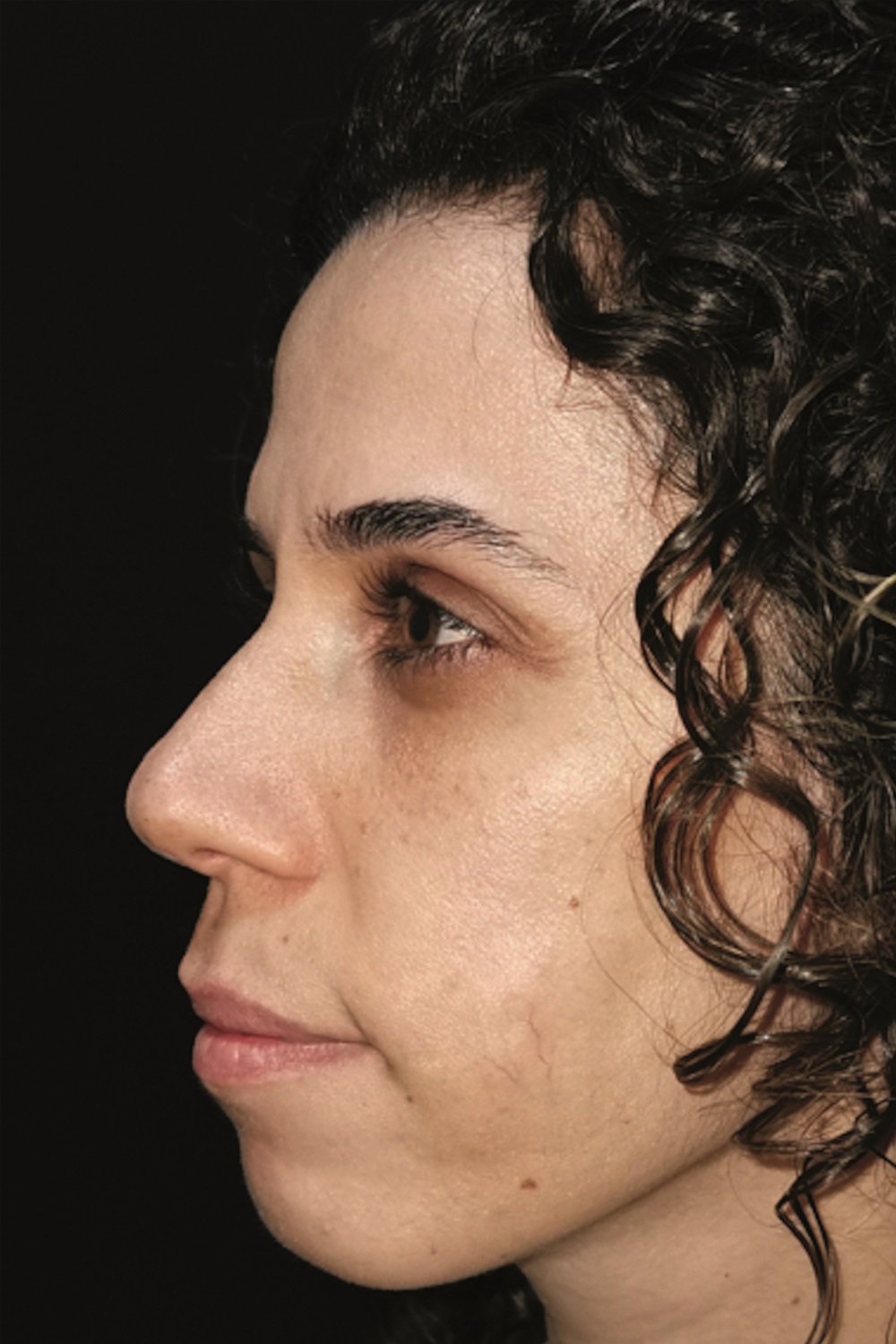

Supplement: ojaa035_suppl_Supplementary_Figure_3H [file ojaa035_suppl_Supplementary_Figure_3H.jpg]
