# Supplementary material for: New Mechanical Fat Separation Technique: Adjustable Regenerative Adipose-tissue Transfer (ARAT) and Mechanical Stromal Cell Transfer (MEST)
Source: Aesthet Surg J Open Forum. 2020 Jul 22;2(4):ojaa035. doi: 10.1093/asjof/ojaa035 (PMC7780457; doi:10.1093/asjof/ojaa035)
Supplement: ojaa035_suppl_Supplementary_Figure_Legend [file ojaa035_suppl_Supplementary_Figure_Legend.docx]

**Supplemental Figure Legend**

**Supplemental Figure 1**. A 49-year-old female patient operated on with the MEST technique IPs1. (A) Preoperative and postoperative frontal view at (B) 6 months, (C) 1 year, and (D) 2 years. (E) Preoperative and postoperative lateral view at (F) 6 months, (G) 1 year, and (H) 2 years.

**Supplemental Figure 2**. A 28-year-old female patient operated on with the ARAT and MEST technique IPs3 after obesity surgery. (A) Preoperative and postoperative frontal view at (B) 6 months, (C) 1 year, and (D) 2 years. (E) Preoperative and postoperative lateral view at (F) 6 months, (G) 1 year, and (H) 2 years.

**Supplemental Figure 3**. A 21-year-old female patient operated on with the ARAT and MEST technique IPs2 for soft tissue reconstruction of her left cheek. She had unsuccessful fat grafting history 2 years before the procedure. A total of 12 cc fat was used. (A) Preoperative and postoperative frontal view at (B) 6 months, (C) 1 year, and (D) 2 years. (E) Preoperative and postoperative lateral view at (F) 6 months, (G) 1 year, and (H) 2 years.
